# CORO1A: a pan-cancer prognosis, diagnostic and immune biomarker based on breast cancer validation

**DOI:** 10.3389/fonc.2025.1670526

**Published:** 2025-10-06

**Authors:** Dilraba Elihamu, Yongxiang Li, Yiyang Wang, Haiyan Cui, Yiting Xing, Haohao Peng, Dilimulati Ismtula, Chenming Guo

**Affiliations:** ^1^ Department of Breast Surgery, Center of Digestive and Vascular Surgery, The First Affiliated Hospital of Xinjiang Medical University, Urumqi, China; ^2^ Thyroid and Breast Surgery Department of the People’s Hospital of Bayingolin Mongol Autonomous Prefecture, Korla, China

**Keywords:** CORO1A, prognosis, tumor immunity, pan-cancer, breast cancer

## Abstract

**Introduction:**

CORO1A, a constituent of the Coronins family, is a conserved protein throughout evolution that interacts with actin within the cellular environment besides being involved in various malignancy development.

**Methods:**

A comprehensive analysis examining the discrepancies in expression levels, survival outcomes, immune cell infiltration (ICI), and enrichment profiles was performed. The expression of CORO1A in breast cancer tissues was verified by immunohistochemistry, WB and RT-qPCR experiments. The effects of different expression levels of CORO1A on the growth and metastasis of breast cancer cell lines were verified by CCK-8, colony formation, Trasnwell and cell scratch experiments.

**Results:**

The findings revealed a heightened expression of CORO1A in the 66.7% of tumor types (22/33), CORO1A consistently demonstrates high diagnostic potential and variable prognostic significance across cancers. In particular, its diagnostic value in SKCM reaches as high as 98%, with a prognostic hazard ratio of 0.77. CORO1A often characterized by hypomethylation of its promoter region, which correlates with the ICI level. Enrichment analysis highlights the critical contribution of CORO1A in B cell receptor pathways and other immune-linked processes, CORO1A may have multiple potential roles in B-cell receptor pathways, involving aspects such as signal transduction regulation, cytoskeletal remodeling and migration, as well as interactions with other immune molecules. exerting a substantial influence on patient prognosis. CORO1A is highly expressed in breast cancer tissues. Breast cancer patients with high expression of CORO1A have a good prognosis. CORO1A knockdown inhibits the growth and metastasis of breast cancer cells, while overexpression is the opposite. Therefore, due to its high diagnostic sensitivity and prognostic value demonstrated in various cancers, CORO1A holds promise as a candidate molecule for novel targeted therapies.

**Discussion:**

CORO1A, as a promising research target, has demonstrated significant and specific capabilities in predicting disease prognosis, analyzing immune responses, and exploring therapeutic approaches for different types of tumors, further highlighting the necessity for in-depth research on it.

## 1 Introduction

According to the latest estimates by the International Agency for Research on Cancer (IARC), there were nearly 20 million new cancer cases in 2022, with 9.7 million deaths from cancer (1). Cancer is a significant worldwide public health issue, defined by the uncontrolled growth and spread of malignant cells that evade inhibition and promote invasion and metastasis (2). Although there is some understanding of cancer pathogenesis, the specific mechanisms remain unclear (3). Due to the diverse biological characteristics of tumor cells, identifying effective molecular markers is crucial for determining their occurrence and progression (4, 5). Prior studies have highlighted that tumor cells have an interaction with stromal cells, which is pivotal in tumor development (6, 7). While cancer research has made strides in understanding tumor biology, the role of specific cytoskeletal regulators like CORO1A remains underexplored. This study will delve into the role of CORO1A in cancer.

The CORO1A, a conserved member of the Coronins family, binds to F-actin and is integral to cytoskeletal remodeling (8, 9), which is influenced by extracellular signals to regulate migration, phagocytosis, and cell polarization (10, 11). The CORO1A possesses a significant implication in tumor development. Although its importance in cancers such as breast cancer (BRCA) (12), thymoma (THYM) (13), and cutaneous melanoma (14) is well-documented, additional research is necessitated to understand its function across various cancer types. This study provides a pan-cancer analysis of CORO1A, integrating insights from expression profiles, methylation patterns, and immune interactions to explore its multifaceted role in tumorigenesis.

This research endeavor delves into the pan-cancer expression profile, diagnostic utility, and prognostic relevance of CORO1A by leveraging an online database platform. It extends its scope to scrutinize the methylation patterns and genetic variations of CORO1A, evaluating their interplay with the immune response, the immune cell infiltration (ICI), and the immune-linked gene expression. Furthermore, the study incorporates functional and metabolic pathway enrichment analyses to bolster future investigative efforts aimed at unraveling the CORO1A functional dynamics in cancer biology.

## 2 Materials and methods

### 2.1 Data download and CORO1A expression differences analysis

The clinical data and RNA sequencing (RNA-seq) for a comprehensive pan-cancer cohort consisting of 15,776 subjects were obtained from the UCSC XENA platform (September 11, 2024; https://xenabrowser.net/datapages/). This data amalgamates insights from the Cancer Genome Atlas (TCGA) and the Genotype-Tissue Expression (GTEx) project, providing a robust foundation for pan-cancer research.

### 2.2 Determine the diagnostic and prognostic capabilities of CORO1A

Samples were categorized into low and high-expression groups employing the median level of CORO1A mRNA expression. Afterward, the link between CORO1A mRNA expression and patient prognosis was determined utilizing Cox regression analysis, including overall survival (OS), disease-specific survival (DSS), and progression-free survival (PFS). The Cox regression and Kaplan-Meier (KM) analyses were employed with the ‘Survival’ and ‘survminer’ packages. Moreover, ggplot2 was utilized to visualize our results using forest plots and Venn diagrams.

The KM Plotter is an online database (September 11, 2024; https://kmplot.com/analysis/) that conducts meta-analyses of gene prognostic values. For further analysis, participants were allocated into two high and low-expression groups—employing the median level of the CORO1A gene probe mRNA expression (200696_s_at).

The “pROC” package was deployed to Receiver Operating Characteristic curve (15) analysis, and “ggplot2” was utilized for visualizing the results. An AUC of 0.7 to 0.9 implies that CORO1A has moderate diagnostic potential, whereas an AUC above 0.9 signifies strong diagnostic capability.

### 2.3 Development and calibration of nomograms

Univariate and multivariate Cox regression analyses were conducted to estimate risk variables affecting patient prognosis. Variables with *p* < 0.05 were chosen for multivariate Cox analysis. The CORO1A expression levels were divided into low and high groups according to the median and were considered independent variables. The prognostic nomogram constructed in this study aims to quantify the independent impact of CORO1A expression levels on the prognosis of patients with breast cancer and others, and integrates other key clinical variables to provide a tool for individualized prognostic assessment that visualizes the prediction of patients’ 1-year, 3-year, and 5-year survival probabilities, and its predictive validity was assessed utilizing the concordance index (C-index) with 1,000 iterations. Calibration curves were used to compare projected and actual results. In constructing the multivariable Cox proportional hazards model using stepwise regression, we set the significance level for variable selection at p < 0.1 to reduce the risk of excluding potentially important predictive factors (Type II error). However, for the variables in the final model, their statistical significance is interpreted using a standard of p < 0.05.

### 2.4 Use of online databases

The TISIDB (September 11, 2024; http://cis.hku.hk/TISIDB/index.php) database’s ‘Isotype’ module was employed to examine the link between CORO1A expression and various human cancer subtypes, emphasizing the association between methylation levels and ICI (16).

The TCGA module within the UALCAN database serves to compare the CORO1A gene promoter methylation levels between normal tissue samples and those from the TCGA (September 11, 2024; http://ualcan.path.uab.edu/index.html). For an in-depth analysis of protein levels across various cancers and their adjacent normal tissues, the CPTAC (Clinical Proteomic Tumor Analysis Consortium) was employed (17).

Afterward, CNV% is evaluated in the ‘Mutations’ module of the Gene Set Cancer Analysis (GSCA) database (September 11, 2024; https://guolab.wchscu.cn/GSCA/#/), examines the association between CORO1A expression, Analysis of the effects of CORO1A methylation and CNV alterations on pan-cancer prognoses based on CORO1A methylation and CNV changes (18).

The “OncoPrint” module of the cBioPortal database (September 11, 2024; https://www.cbioportal.org) was deployed to analyze the CORO1A genetic alterations level in the “TCGA Pan-Cancer Atlas Study” dataset (10,443 samples having mutation data across 32 studies) (19). The “Cancer Type Summary” module evaluates alterations in CORO1A, the number of gene mutations, the kind of mutations, and the recurrence of CNV for each type of cancer. The mutation sites of CORO1A were assessed using the “mutagenesis” module and shown in the three-dimensional structure of their proteins.

The proportion of CORO1A for each CNV and single nucleotide variant (SNV) type in pan-cancer is downloaded from the Cancer Somatic Mutation Directory (COSMIC) (September 11, 2024; https://cancer.sanger.ac.uk/cosmic).

### 2.5 Correlation between CORO1A and tumor immunity

Herein, we conducted an investigation into the correlation between the gene CORO1A and two key cancer biomarkers—tumor mutational burden (TMB) and microsatellite instability (MSI)—across a spectrum of malignancies, utilizing the Sangerbox 3.0 online database for our analysis (September 11, 2024; http://vip.sangerbox.com/). The ESTIMATE algorithm, along with the ‘GSVA’ and ‘org.Hs.eg.db’ tools, was employed to calculate the Stromal, Immune, and ESTIMATE Scores (20). Additionally, the correlation between CORO1A expression and eight immune checkpoint-linked gene transcripts was explored in different cancer types. A gene list comprising immune activators, immunosuppressives, chemokines, chemokine receptors, and Gene Set Enrichment Analysis (GSEA) was deployed to analyze gene set enrichment and major histocompatibility complex (MHC) molecules (September 11, 2024; https://www.gsea-msigdb.org/gsea/msigdb/index.jsp). The correlation between CORO1A and immune-linked gene expression was ascertained with the Spearman correlation coefficient.

A methodology was developed to estimate the ICI of 24 immune cells across different malignancies with the single-sample GSEA (ssGSEA) algorithm (21). The correlation between CORO1A gene expression and the various ICIs was elucidated in a spectrum of cancers. To achieve this, the EPIC, TIMER, CIBERSORT, and MCPCOUNTER methods were employed, which are integrated within the ‘Immune’ module of the TIMER2.0 database (September 11, 2024; http://timer.cistrome.org/). Our comprehensive analysis encompassed a diverse range of immune cell populations, including cancer-associated fibroblasts (CAFs), CD8+ and CD4+ T cells, regulatory T cells (Tregs), B cells, neutrophils, monocytes, myeloid dendritic cells (mDCs), macrophages, and natural killer (NK) cells (22).

### 2.6 Functional and pathway enrichment analysis

Gene Ontology (GO) and Kyoto Encyclopedia of Genes and Genomes (KEGG) were utilized to investigate the biological roles and predicted pathways of CORO1A and its associated proteins.

Protein-protein interaction (23) involves proteins interacting to regulate gene expression, transmit biological signals, and control the cell cycle (24). The STRING database(September 11, 2024; https://string-db.org/) includes over 24 million proteins from more than 5,000 organisms, analyzing known and predicted physical and functional protein associations. This database was utilized to construct and visualize the interaction network of CORO1A-related proteins and to investigate their interactions.

### 2.7 Western blot analysis

Twelve pairs of BRCA and surrounding normal tissue samples were procured from the Department of Breast Surgery at the First Affiliated Hospital of Xinjiang Medical University. Total proteins were extracted utilizing RIPA buffer enriched with PMSF protease inhibitor (Beyotime, China). Protein concentrations were quantified with the BCA assay, and the proteins were then used for Western blot analysis. Proteins were separated with a 4%–15% gradient SDS-PAGE and then transferred to PVDF membranes. Membranes were incubated at ambient temperature with TBST containing 5% skim milk for blocking and incubated overnight at 4 °C with primary antibodies. A rabbit and mouse monoclonal antibodies specific to CORO1A (1:1000, Abcam, USA; ab203698) and specific to β-actin (1:8000, Santa Cruz Biotechnology, USA; sc-69879), respectively, were utilized. Following washing with TBST, the PVDF membranes were incubated with either goat anti-rabbit or -mouse IgG (both 1:8000, Cell Signaling Technology, USA. #7074 or #7076) protein bands were observed via an ECL chemiluminescence kit (Cell Signaling Technology, USA. #7003) and recorded with a Bio-Rad imaging system.

The developed bands were analyzed using ImageJ software to analyze the grayscale values of the bands, β-actin as the CORO1A/β-actin ratio to calculate the standardized protein expression level.

### 2.8 Immunohistochemistry staining

Human tissue microarray slides comprising 80 paired BRCA specimens and adjacent normal tissues (BRC1602, Superbiotek, Inc., Shanghai, China) were utilized in this study. Paraffin-embedded tumor samples were sectioned into 6μm thick slices. Standard immunohistochemistry (IHC) was conducted on the tissue array slides using a protein-specific antibody: anti-CorO1a antibody (1:1000 dilution, ab203698, Abcam, USA). **Table 1** lists comprehensive clinicopathological features.

**Table 1:** The relationship between the expression of CORO1A and the clinicopathological characteristics of BC patients.

CORO1A		Number of cases(n=80)	The expression of CORO1A		P
			Low (n=40)	High (n=40)	
Age	>50 years	40	22	18	0.5026
	≤50 years	40	18	22	
Tumor size(cm)	>2	48	19	29	0.8140
	≤2	32	11	21	
T	T I~II	46	18	28	0.0411
	T III	34	22	12	
N	N 0	30	12	18	0.2481
	N 1~3	50	28	22	
TNM	I–II	32	13	19	0.2537
	III	48	27	21	
Survival status	Live	48	19	29	0.0392
	Death	32	21	11	

For the expression level of CORO1A protein, a semi-quantitative scoring system was used to score it: according to the staining intensity, it was divided into 0~3 points (no staining = 0 points, weak positive = 1 point, moderate positive = 2 points, strong positive = 3 points); According to the proportion of positive cells, it is divided into 1~4 points (positive rate≤ 25%=1 point, 26%–50%=2 points, 51%–75%=3 points, ≥76%=4 points). The final composite score was obtained by multiplying the staining intensity by the positive proportion score, and the total score ranged from 0 to 12 points. According to the distribution of scores, the cases were divided into low expression group (0–5 points) and high expression group (6–12 points) for further statistical analysis.

### 2.9 Plasmid construction and transfection

Construction of CORO1A knockdown vector (sh-CORO1A): Three sequences of specific small hairpin RNA (shRNA) (**Supplementary Table S1**) were selected to knock down CORO1A expression, and the shRNA was inserted into the lentiviral vector pLKO.1-puro. Cloning was performed by restriction enzyme digestion and ligation reactions, and the constructed plasmids were verified by DNA sequencing. Construction of CORO1A overexpression vector: Lentiviral vector pLVX-Puro was used to insert the CORO1A gene into the appropriate position and select the appropriate promoter to drive the expression of CORO1A.

### 2.10 The constructed vector was confirmed by DNA sequencing

The constructed shRNA or overexpression plasmid (2 µg) was mixed with the transfection reagent Lipofectamine 3000 (Invitrogen, Shanghai, China) based on the proportion indicated by the reagent and transfected into HEK-293T cells that grown to about 80%. The cells were cultured for 48 h to allow virus particles to be produced, thereby collecting, filtering, and concentrating the supernatant by ultracentrifugation (100,000 x g, 4°C, 2 h). After ultracentrifugation, the supernatant was discarded, and the pellet was resuspended in a medium.

### 2.11 Lentivirus infection and grouping

Breast cancer cell lines MCF-7 and MDA-MB-231 were inoculated in the plates, and virus infection was performed when the cells grew to about 80%. The concentrated lentivirus solution was introduced to the cells, which were replaced with fresh culture medium 24 h after infection and continued to be cultured for 48 h. According to the above methods, the cells were divided into four groups: CORO1A knockdown empty vector group (NC-KD); CORO1A knockdown group (KD); CORO1A overexpression vector group (NC-OE); CORO1A overexpression group (OE). The expression of genes was observed by fluorescence microscopy 72 h post-infection, culturing the cells in good condition for a period and collected for subsequent experiments.

### 2.12 RNA extraction and qRT-PCR

Following the protocols, we extracted total RNA by Trizol (Invitrogen, Shanghai, China) via the SYBR Green PCR kit (Takara, Beijing, China). China) Furthermore, forward and reverse primers (**Table 2**) were used to amplify the target genes in a 20 µL final volume. The qRT-PCR reaction process was performed in Applied Biosystems 7500 (Foster City, CA, USA), and data were analyzed with the 2–ΔΔCT method.

**Table 2:** shRNA sequences and primer sequences for CORO1A.

Name	Targeting sequence (5’→3’)
sh-CORO1A-1	GCAGATCAACATCTTCGAA
sh-CORO1A-2	GGACATGTATGTCGAGAAT
sh-CORO1A-3	GCTGGAGAACTTCAAGATG
CORO1A-OE-F	GGATCCATGGAGTACAGCCTGAGGAG
CORO1A-OE-R	CTCGAGTCAGGTTGTTGCTGTTGTTG
CORO1A-F	ATGGAGTACAGCCTGAGGAG
CORO1A-R	TCAGGTTGTTGCTGTTGTTG
GAPDH-F	GAAGGTGAAGGTCGGAGTC
GAPDH-R	GAAGATGGTGATGGGATTTC

### 2.13 CCK-8 assay

The cell viability assay kit (CCK-8; Dojindo Molecular Technologies, Japan) was used to assess the cell proliferation ability by seeding cells in 96-well plates and culturing them for five days. Subsequently, the CCK-8 reagent was added to each well, followed by incubation at 37°C for 1.5 h. The absorbance value at 450 nm was then measured using a microplate reader (Tecan Infinite, Switzerland). The measured results were plotted as a line chart to reflect the changes in cell proliferation capacity.

### 2.14 Transwell assay

The Transwell assay kit (Corning, New York, USA) was used to seed cells from the experimental and control groups into Transwell chambers for invasion and migration assays, with or without Matrigel in the chambers. A 600 µL volume of 10% FBS medium was added to the lower chamber as a chemoattractant, followed by incubation for 24 h at 37°C in 5% CO_2_. Residual cells on the upper surface of the Transwell membrane were removed with a cotton swab. Cells that had migrated to the lower chamber were then fixed with 4% paraformaldehyde for half an hour and stained with 0.1% crystal violet (both from Beyotime, Shanghai, China). Finally, cell counts and observations were performed under an inverted microscope (Mshot, Guangzhou, China) at 200× magnification.

### 2.15 Scratch assay

To assess cell migration, scratch assays were performed. Cells were seeded into 6-well plates and cultured to form a confluent monolayer. A sterile 200 µL pipette tip was used to create a uniform scratch. After removing detached cells with PBS, cells were cultured in serum-free medium to reduce proliferation. Images of the scratched areas were taken at 0 and 24 h using an inverted microscope (Leica, Germany) at 100× magnification. The scratch area was measured with ImageJ, and migration rate was calculated as (initial scratch area - final scratch area)/initial scratch area.

### 2.16 Statistical analysis

Statistical analyses were conducted with R software (version 4.0.3, https://www.R-project.org/). For data visualization, we employed the ‘ggplot2’ package. The Mann-Whitney U test was deployed to assess the variability in CORO1A expression levels among unmatched samples, while the Wilcoxon signed-rank test was used for paired samples. Additionally, the Spearman correlation coefficient was utilized to explore the correlations between CORO1A expression levels and several factors, including m6A methylation regulators, TMB, MSI, immune scores, and immune-associated genes. A threshold of *p* < 0.05 was set to determine statistical significance. When calculating the hazard ratio (HR), a 95% confidence interval was employed. AND, to control for false positives due to multiple comparisons, we applied the Benjamini-Hochberg procedure to differentially expressed gene analysis to correct p-values. The significance threshold is set at q<0.05 (i.e., FDR<5%).

## 3 Results

### 3.1 Differences in pan-cancer and its subforms of CORO1A expression and protein content

**Figure 1:**
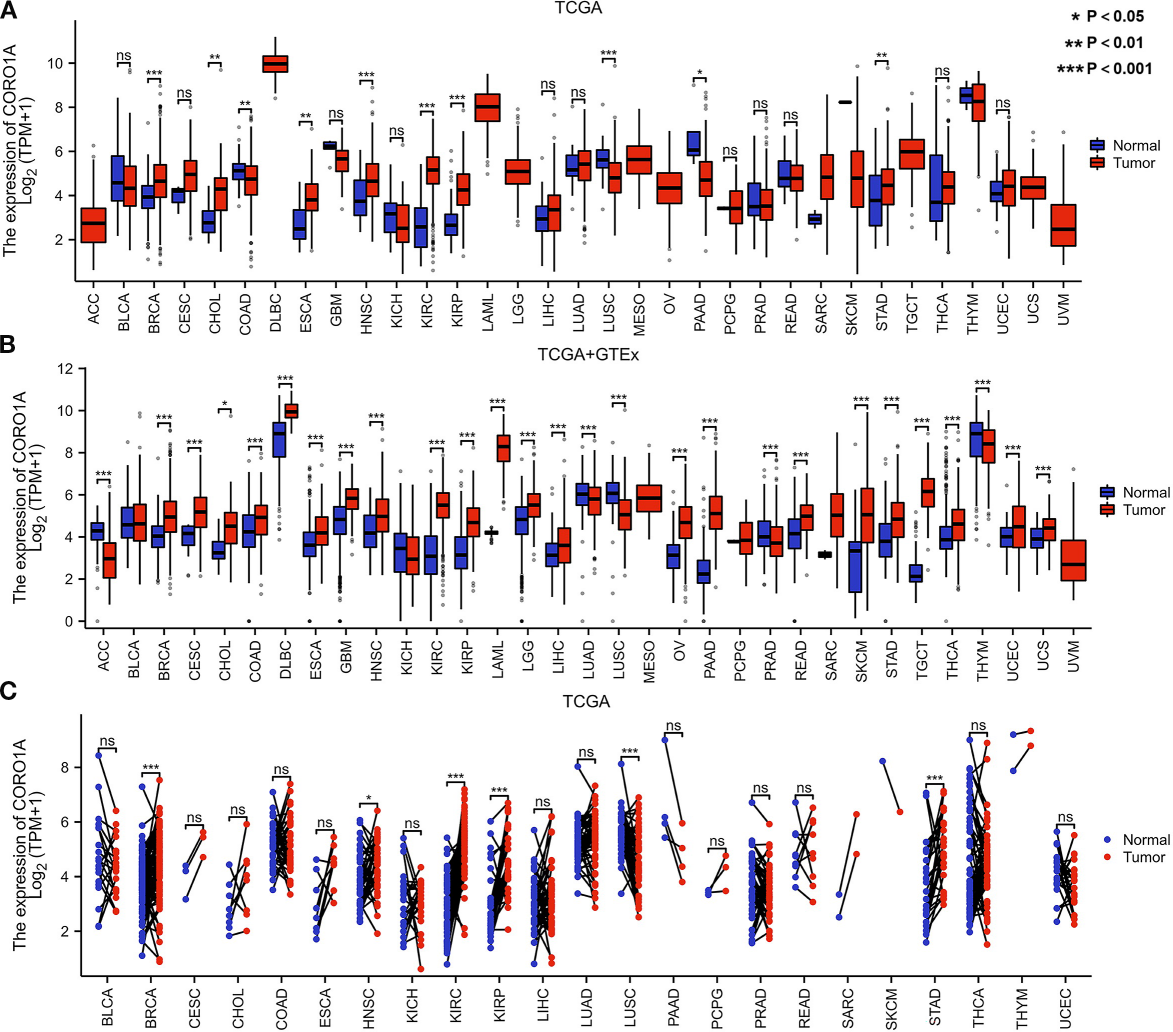
Differential CORO1A expression in 33 cancers. **(A)** CORO1A mRNA expression difference between TCGA and **(B)** GTEx tumor and normal tissues. **(C)** CORO1A mRNA expression in TCGA tumor and paired normal tissues.

Analysis of the TCGA database revealed significantly reduced CORO1A mRNA expression in tumor tissues of colon adenocarcinoma [COAD, P = 0.006, (95% CI) = (-0.670 – -0.113)], lung squamous cell carcinoma [LUSC, P<0.001, (95% CI) = (-1.051 – -0.541)], and pancreatic cancer [PAAD, P = 0.010, (95% CI) = (-3.391 – -0.418)], unlike normal tissues (P < 0.05). The tumor tissues exhibited significantly greater expression levels than their normal counterparts in several cancer forms, including Breast Cancer [BRCA, P<0.001, (95% CI) = (0.578 – 0.971)], cholangiocarcinoma [CHOL, P = 0.007, (95% CI) = (0.398 – 2.165)], esophageal cancer [ESCA, P = 0.001, (95% CI) = (0.544 – 1.829)], head and neck squamous cell carcinoma [HNSC, P<0.001, (95% CI) = (0.456 – 1.156)], renal clear cell carcinoma [KIRC, P<0.001, (95% CI) = (2.293 – 2.812)], renal papillary cell carcinoma [KIRP, P<0.001, (95% CI) = (1.037 – 1.846)], and gastric cancer [STAD, P = 0.005, (95% CI) = (0.219 – 1.210)] (P < 0.05; **Figure 1A**).

Moreover, we supplemented limited normal tissue data for specific tumors and normal samples from the TCGA and GTEx databases to improve the reliability of our outcomes. The study revealed significantly reduced CORO1A mRNA expression in adrenocortical carcinoma [ACC, P<0.001, (95% CI) = (-1.601 – -1.012)], lung adenocarcinoma [LUAD, P<0.001, (95% CI) = (-0.439 – -0.193)], [LUSC, P<0.001, (95% CI) = (-1.185 – -0.926)], prostate cancer (25), and Thymoma [THYM, P<0.001, (95% CI) = (-0.574 – -0.182)] in comparison to normal tissues (P < 0.05). Conversely, its expression manifested a significant rise in 22 other malignant tumors, including breast [BRCA, P<0.001, (95% CI) = (0.835 – 1.089)], cervical [CESC, P<0.001, (95% CI) = (0.733 – 1.828)], [CHOL, P = 0.012, (95% CI) = (0.314 – 2.057)], [COAD, P<0.001, (95% CI) = (0.454 – 0.802)], diffuse large B-cell lymphoma [DLBC, P<0.001, (95% CI) = (0.871 – 1.399)], esophageal [ESCA, P<0.001, (95% CI) = (0.462 – 0.773)], glioblastoma [GBM, P<0.001, (95% CI) = (0.902 – 1.181)], [HNSC, P<0.001, (95% CI) = (0.365 – 1.083)], [KIRC, P<0.001, (95% CI) = (2.153 – 2.641), renal papillary cell [KIRP, P<0.001, (95% CI) = (1.098 – 1.785)], acute myeloid leukemia [LAML, P <0.001, (95% CI) = (3.954 – 4.323)], low-grade glioma [LGG, P<0.001, (95% CI) = (0.648 – 0.839)], liver [LIHC, P<0.001, (95% CI) = (0.214 – 0.606)], ovarian [OV, P<0.001, (95% CI) = (1.267 – 1.742)], pancreatic [PAAD, P<0.001, (95% CI) = (2.518 – 2.960)], rectal [READ, P<0.001, (95% CI) = (0.545 – 1.018)], [SKCM, P<0.001, (95% CI) = (2.042 – 2.408)], gastric [STAD, P<0.001, (95% CI)= (0.759 – 1.139)], testicular germ cell [TGCT, P<0.001, (95% CI) = (3.682 – 4.082)], thyroid [THCA, P<0.001, (95% CI) = (0.438 – 0.730)], endometrial [UCEC, P<0.001, (95% CI) = (0.245 – 0.779)], and urothelial carcinoma [UCS, P <0.001, (95% CI) = (0.234 – 0.757)] (P < 0.05; **Figure 1B**).

**Figure 2:**
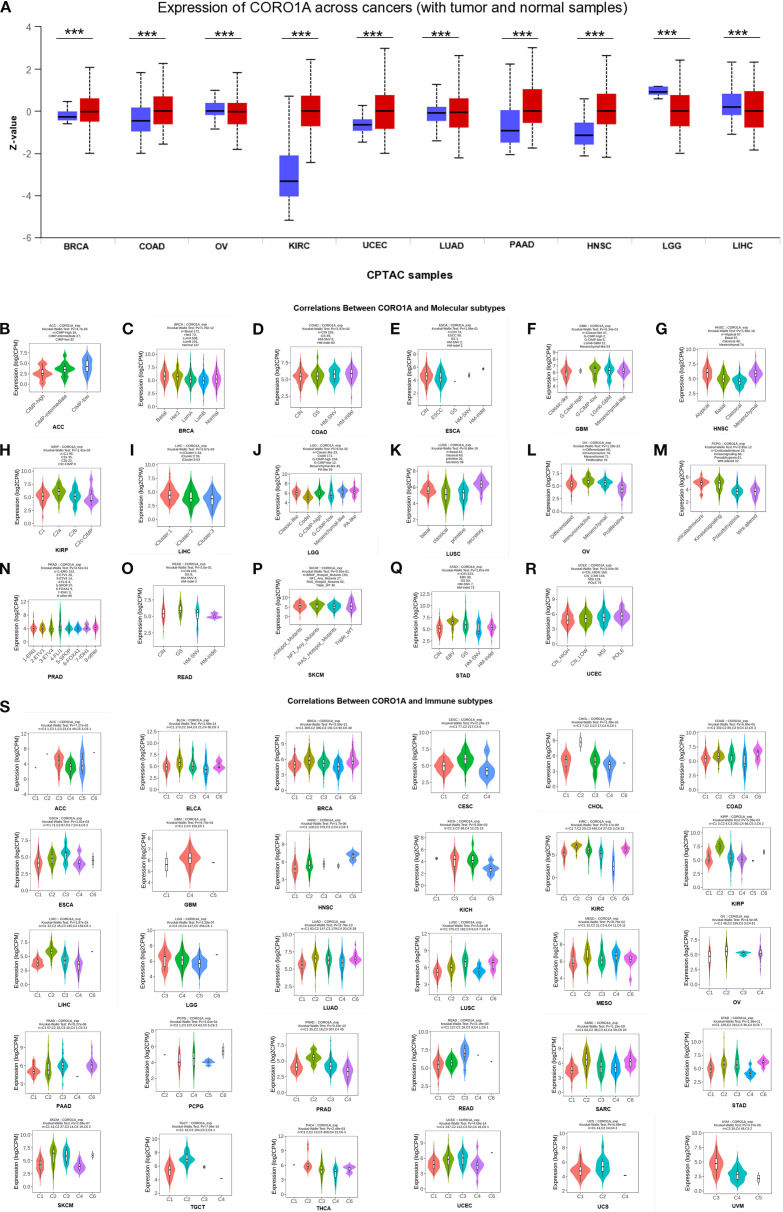
Variations in CORO1A protein content levels in pan-carcinoma. **(A)** Variations in CORO1A protein levels in pan-carcinoma, encompassing BRCA, COAD, OV, KIRC, UCEC, LUAD, PAAD, HNSC, LGG, and LIHC. Connections between molecular subtypes and CORO1A expression in TCGA cancers, encompassing **(B)** ACC; **(C)** BRCA; **(D)** COAD; **(E)** ESCA; **(F)** GBM; **(G)** HNSC; **(H)** KIRP; **(I)** LIHC; **(J)** LGG; **(K)** LUSC; **(L)** OV; **(M)** PCPG; **(N)** PRAD; **(O)** READ; **(P)** SKCM; **(Q)** STAD; **(R)** UCEC. **(S)** Links between immune subtypes and GSN expression in TCGA tumors, including ACC, BLCA, BRCA, CESC, CHOL, COAD, ESCA, GBM, HNSC, KICH, KIRC, KIRP, LGG, LIHC, LUAD, LUSC, MESO, OV, PAAD, PCPG, PRAD, READ, SARC, SKCM, STAD, TGCT, THCA, UCEC, UCS, and UVM. C1 (wound healing), C2 (IFN-g dominant), C3 (inflammatory), C4 (lymphocyte deplete), C5 (immunologically quiet), and C6 (TGF-b dominant). ***p<0.001.

Only LUSC malignant tissue from paired samples of 23 malignancies exhibited significantly lower CORO1A mRNA expression when compared to adjacent tissues. The malignant tissues encompassing BRCA [P<0.001, (95% CI) = (0.264-0.709)], HNSC [P = 0.049, (95% CI) = (0.001 -0.848)], KIRC [P<0.001, (95% CI) = (2.246 – 2.987)], KIRP [P<0.001, (95% CI) = (1.011–2.139)], and STAD [P<0.001, (95% CI) = (0.715 – 1.886)] experienced significantly elevated CORO1A mRNA expression (P < 0.05; **Figure 1C**). Utilizing the UALCAN database, we manifested that the cancer tissues like OV, LUAD, GBM, and LIHC revealed mitigated CORO1A protein levels compared to normal tissues, but increased in cancer types encompassing BRCA, COAD, HNSC, KIRC, PAAD, and UCEC (P < 0.05; **Figure 2A**). Additionally, the TISIDB database was employed to examine CORO1A expression across different pan-cancer immune and molecular subtypes. Moreover, CORO1A is differentially expressed in 16 cancer subtypes (**Figures 2B–R**). High CORO1A expression was identified in specific subtypes across various cancers: HER-2 in BRCA (**Figure 2B**), HM-indel in COAD (**Figure 2C**), CIN in ESCA (**Figure 2D**), G-CIMP-low in GBM (**Figure 2E**), Acute in HNSC (**Figure 2F**), C2a in KIRP (**Figure 2G**), Mesenchymal-like in LGG (**Figure 2H**), iCluster 1 in LIHC (**Figure 2I**), Immunoreactive in OV (**Figure 2J**), GS in READ (**Figure 2K**), NF1-Any-Mutants in SKCM (**Figure 2L**), EBV in STAD (**Figure 2M**), and POLE in UCEC (**Figure 2N**). Conversely, low CORO1A expression was noted in the CIMP-low subtype of ACC (**Figure 2O**), secretory subtype of LUSC (**Figure 2P**), and 4-FLI1 subtype of PRAD (**Figure 2Q**). Furthermore, CORO1A expression in PCPG was insignificant and excluded from further analysis (**Figure 2R**). In addition, CORO1A expression significantly correlated with immune subtypes across 30 cancers, peaking in the C2 (inflammatory) and nadir in the C4 (lymphocyte depletion) subtypes (**Figure 2S**).

### 3.2 Predictive and diagnostic importance of CORO1A in different cancer types

**Figure 3:**
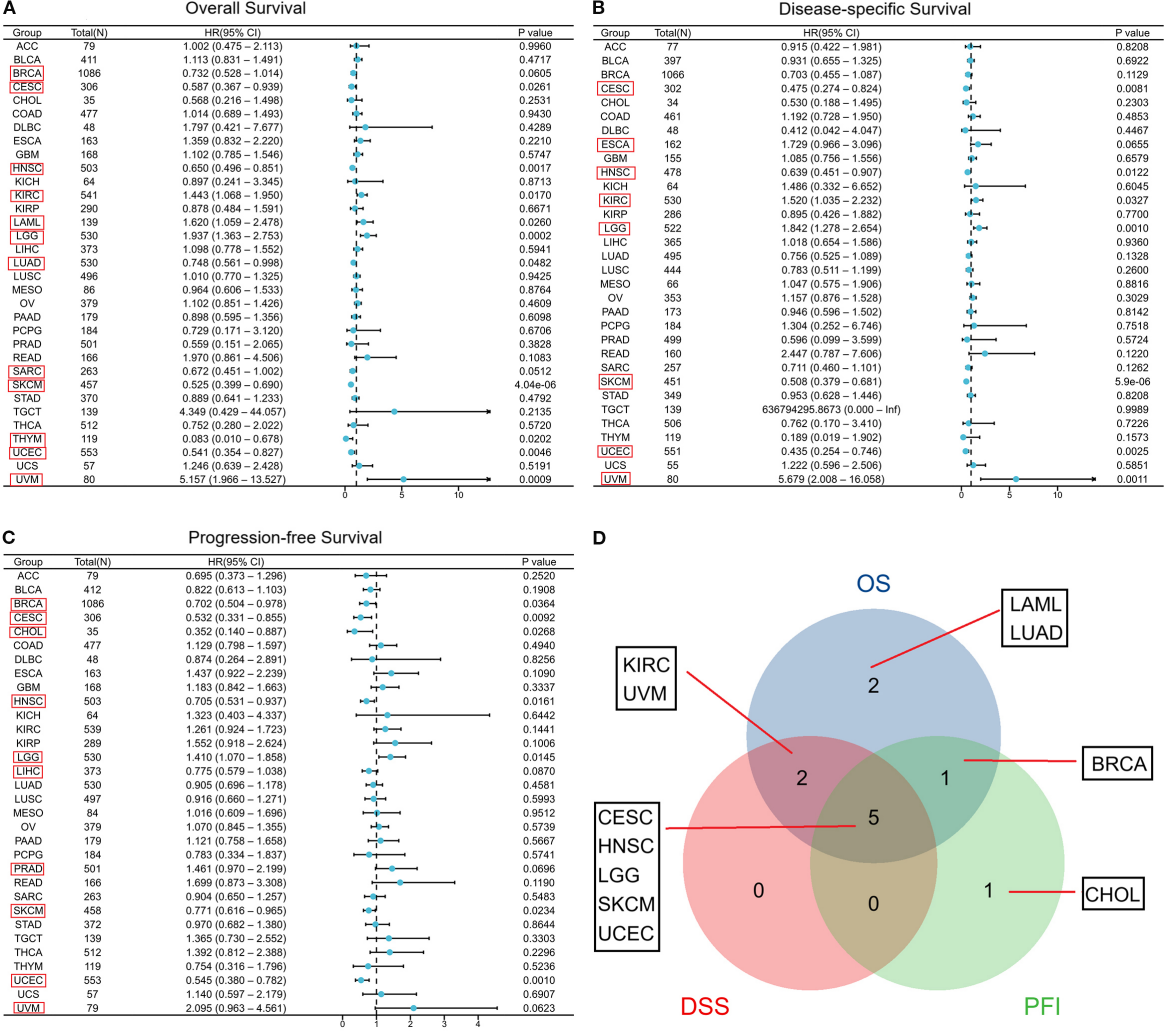
Connection between CORO1A expression and prognosis in cancer patient prognosis. Correlation between CORO1A expression and OS **(A)**, DSS **(B)**, and PFS **(C)** in cancer patients. **(D)** The Venn diagram illustrates the intersection of OS, DS, and PFS for various cancers.(Among them, we included seven types of cancer that had a p<0.05 in univariate COX regression analysis for both univariate and multivariate regression analysis, which are marked in red frames in the forest plot C.).

**Figure 4:**
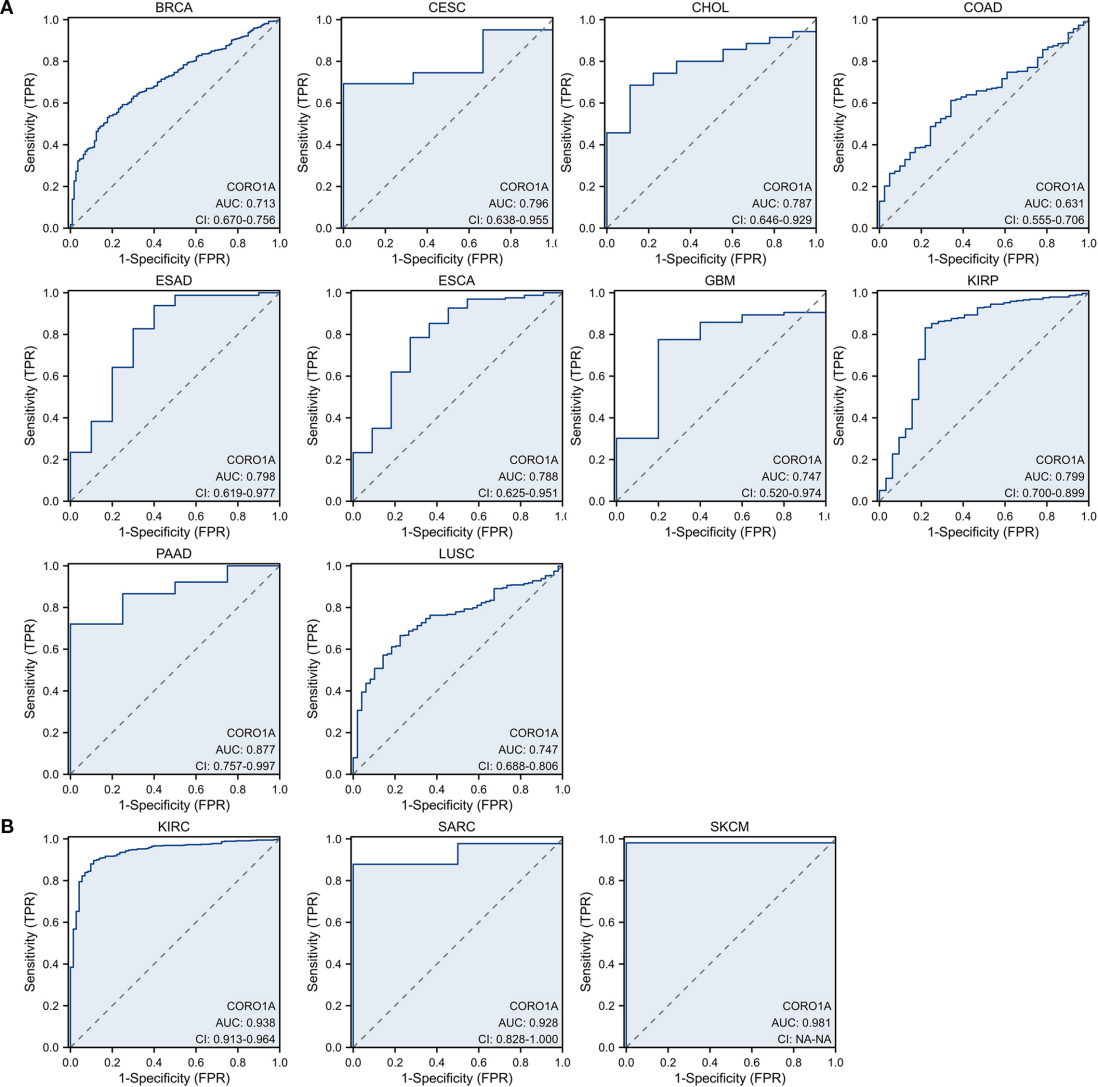
Receiver operating characteristic ^[14]^ curve of CORO1A expression in pan-carcinoma. CORO1A expresses cancer with several diagnostic significance (AUC > 0.7), encompassing BRCA, CESC, CHOL, ESAD, ESCA, GBM, KIRP, PAAD, and LUSC **(A)**, and with good diagnostic value (AUC > 0.9), encompassing KIRC, SARC, and SKCM **(B)**.

Here, we evaluated the predictive significance of CORO1A, focusing on OS, DSS, and PFS using the TCGA database. Moreover, we included cancers with P < 0.1 in our analysis. High CORO1A expression revealed the link to enhanced OS in BRCA [P = 0.061, HR (95% CI) = 0.732 (0.528–1.014)], CESC [P = 0.026, HR (95% CI) = 0.587 (0.367 – 0.939)], HNSC [P = 0.002, HR (95% CI) = 0.650 (0.496 – 0.851)], LUAD [P = 0.048, HR (95% CI) = 0.748 (0.561 –0.998)], SKCM [P<0.001, HR (95% CI) = 0.525 (0.399–0.690)], and UCEC [P = 0.005, HR (95% CI) = 0.541 (0.354 – 0.827)] but demonstrated an association with mitigated OS in KIRC [P = 0.017, HR (95% CI) = 1.443 (1.068 – 1.950)], LAML [P = 0.026, HR (95% CI) = 1.620 (1.059–2.478)], LGG [P<0.001, HR (95% CI) = 1.937 (1.363–2.753)], THYM [P = 0.020, HR (95% CI) = 0.083 (0.010 – 0.678)] and UVM [P<0.001, HR (95% CI) = 5.157 (1.966 – 13.527)]. It expression in SARC [P = 0.051, HR (95% CI) = 0.672 (0.451–1.002)] was not significant (**Figure 3A**; **Supplementary Figure S1A**). For DSS, high CORO1A expression served as a protective factor for longer DSS in CESC [P = 0.008, HR (95% CI) = 0.475 (0.274–0.824)], HNSC [P = 0.012, HR (95% CI) = 0.639 (0.451 – 0.907)], SKCM [P<0.001, HR (95% CI) = 0.508 (0.379 – 0.681)], and UCEC [P = 0.003, HR (95% CI) = 0.435 (0.254 – 0.746)], while it was a risk factor for reduced DSS in ESCA [P = 0.066, HR (95% CI) = 1.729 (0.966 – 3.096)], KIRC [P = 0.033, HR (95% CI) = 1.520 (1.035–2.232)], LGG [P = 0.001, HR (95% CI) = 1.842 (1.278 – 2.654)] and UVM [P = 0.001, HR (95% CI) = 5.679 (2.008 – 16.058)] (**Figure 3B**; **Supplementary Figure S1B**). Elevated CORO1A expression is linked to extended PFS in BRCA [P = 0.036, HR (95% CI) = 0.702 (0.504 – 0.978)], CESC [P = 0.009, HR (95% CI) = 0.532 (0.331 – 0.855)], CHOL [P = 0.027, HR (95% CI) = 0.352 (0.140 – 0.887)], HNSC [P = 0.016, HR (95% CI) = 0.705 (0.531 – 0.937)], LIHC [P = 0.087, HR (95% CI) = 0.755 (0.579 – 1.038)], PRAD [P = 0.069, HR (95% CI) = 1.461 (0.970 – 2.199)], SKCM [P = 0.023, HR (95% CI) = 0.771 (0.616 – 0.965)], and UCEC [P = 0.001, HR (95% CI) = 0.545 (0.380 –0.782)] patients, while it is associated with reduced PFS in LGG [P = 0.015, HR (95% CI) = 1.410 (1.070 – 1.858)] patients. CORO1A expression in UVM [P = 0.062, HR (95% CI) = 2.095 (0.963 – 4.561)]was considered insignificant and excluded from analysis (**Figure 3C**; **Supplementary Figure S1C**). Venn diagram analysis elucidated that CORO1A influences three critical prognostic indicators: OS, DSS, and PFS—across five cancer types: CESC, HNSC, LGG, SKCM, and UCEC, highlighting its potential as a key prognostic factor in these cancers (**Figure 3D**). Furthermore, in pan-cancer, we analyzed the CORO1A diagnostic significance, and the outcomes exhibited that CORO1A was effective in BRCA (AUC = 0.713), CESC (AUC = 0.796), CHOL (AUC = 0.787), ESAD (AUC = 0.798), ESCA (AUC = 0.788), GBM (AUC = 0.747), KIRP (AUC = 0.799), PAAD (AUC = 0.877) and LUSC (AUC = 0.747) (AUC > 0.7; **Figure 4A**). It has good diagnostic ability (AUC > 0.9) in three cancers: KIRC (AUC = 0.938), SARC (AUC = 0.988), and SKCM (AUC = 0.98); (**Figure 4B**). In addition, our results show that CORO1A exhibits the best diagnostic efficacy in differentiating SKCM patients from normal controls among the above cancer types.

### 3.3 CORO1A serves as an independent predictive factor in specific cancers

Herein, we conducted a single-variate distribution and multivariate regression analyses on seven cancer types to identify risk factors that influence the PFS of cancer patients. In this paper, we included cancer types with univariate COX regression analysis p < 0.05: BRCA, CESC, CHOL, HNSC, LGG, SKCM, and UCEC. Multivariate analysis identified independent predictors for various cancers: For BRCA, M stage [M1, HR (95% CI) = 3.777 (1.864-7.654), p < 0.001] influenced PFI (**Supplementary Table S1A**). Significant factors in CESC included primary treatment outcome [PR/CR, HR (95% CI) = 0.226 (0.084-0.605), p = 0.003] and clinical stage [III/IV, HR (95% CI) = 0.186 (0.036-0.973), p = 0.046] (**Supplementary Table S1B**). The primary treatment outcome [PR/CR, HR (95% CI) = 0.151 (0.095-0.239), p < 0.001]and N stage [N2/N3,HR (95% CI) = 1.719 (1.140-2.592), p = 0.010] were significant predictors in HNSC (**Supplementary Table S1C**). Age [> 40, HR (95% CI) = 1.876 (1.355-2.597), p < 0.001] and IDH status [Mut, HR(95% CI) = 0.249 (0.170-0.363), p < 0.001] were significant in LGG (**Supplementary Table S1D**). In SKCM (**Supplementary Table S1E**), predictive factors included T [T3/T4, HR(95% CI) = 1.487 (1.101-2.009), p = 0.010], N (N2/N3, HR(95% CI) = 1.584 (1.050-2.390), p = 0.029), pathological stages [III/IV, HR(95% CI) = 1.459 ((1.026-2.073), p = 0.035], and high CORO1A expression [HR(95% CI) = 0.739 (0.564-0.969), p = 0.029]. In UCEC (**Supplementary Table S1F** **),** predictors identified include clinical stage [III/IV, HR(95% CI) = 3.341 (2.262-4.935), p < 0.001], primary treatment outcome (PR/CR, HR(95% CI) = 0.134 (0.082-0.218), *p* < 0.001), and high CORO1A expression [HR(95% CI) = 0.602 (0.403-0.898), p = 0.013].

**Figure 5:**
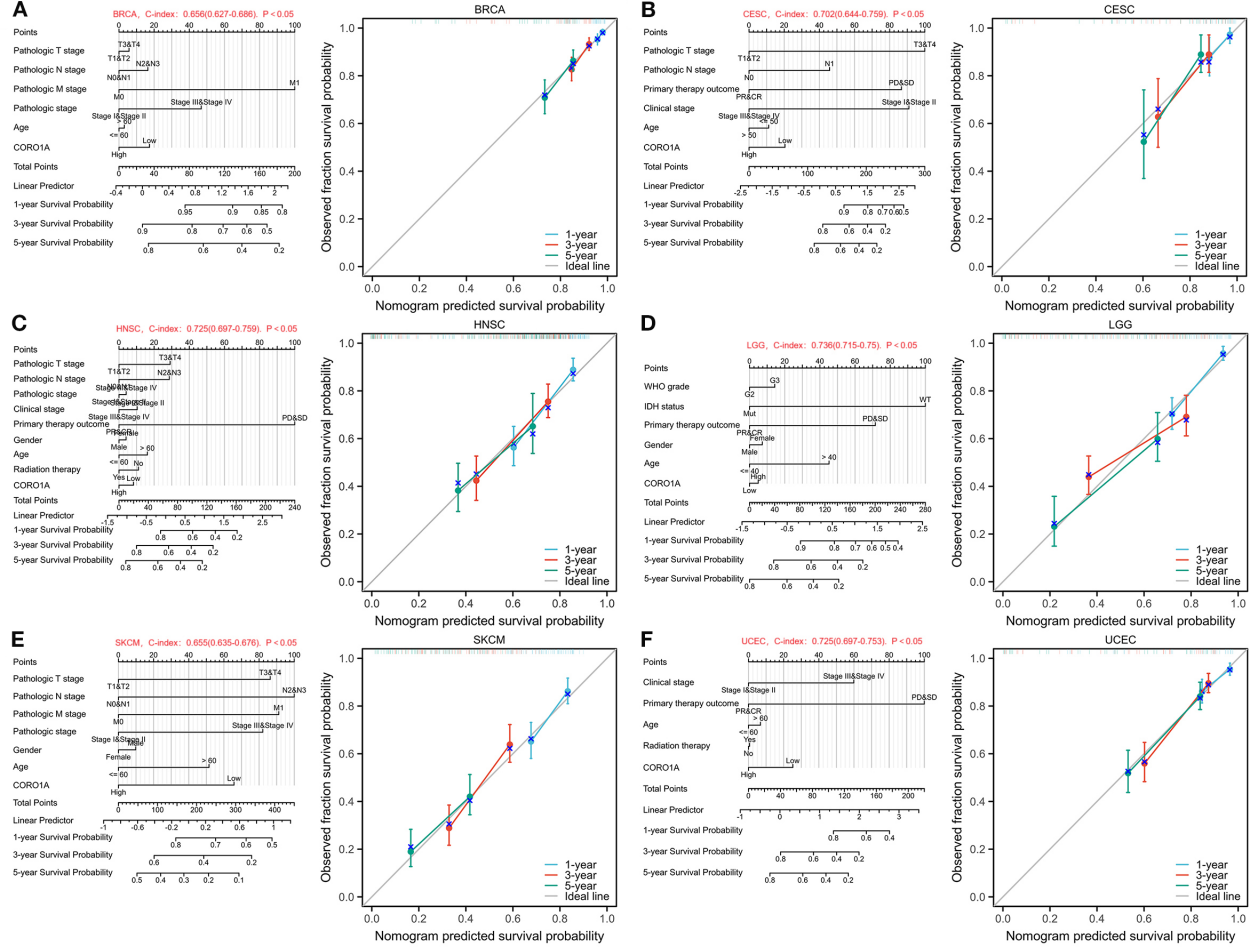
Nomograms and calibration curves predicting patient PFS in six malignancies. Nomograms and calibration curves of BRCA **(A)**, CESC **(B)**, HNSC **(C)**, LGG **(D)**, SKCM **(E)**, and UCEC **(F)**. The horizontal and vertical axes denote the model-predicted and observed survival probability, respectively. The closeness of each line to the optimal line signifies the model’s effectiveness.To use the nomogram, locate the patient’s value for each variable (e.g., RiskScore, Age, Gender, Stage) and draw a line upward to the ‘Points’ axis to determine the corresponding score. Sum the scores for all variables and locate the total on the ‘Total Points’ axis. Finally, draw a line downward from the ‘Total Points’ axis to the survival probability axes to estimate the probability of survival at each time point.”.

We utilize the independent impact of CORO1A expression levels on the prognosis of patients with breast cancer and other conditions, and integrate other key clinical variables to construct prediction nomograms and calibration. The BRCA nomogram achieved a C index of 0.656 (0.627 – 0.686, **Figure 5A**). In CESC, it was 0.702 (0.644 – 0.759, **Figure 5B**), 0.725 (0.697 – 0.752, **Figure 5C**) in HNSC, 0.736 (0.715 – 0.756, **Figure 5D**) in LGG, 0.655 (0.635 - 0.676, **Figure 5E**) in SKCM, and 0.725 (0.697 – 0.753, **Figure 5F**) in UCEC. Subsequently, each nomogram was calibrated to evaluate the model’s reliability.

### 3.4 Differences in CORO1A methylation modification levels in pan-cancer

**Figure 6:**
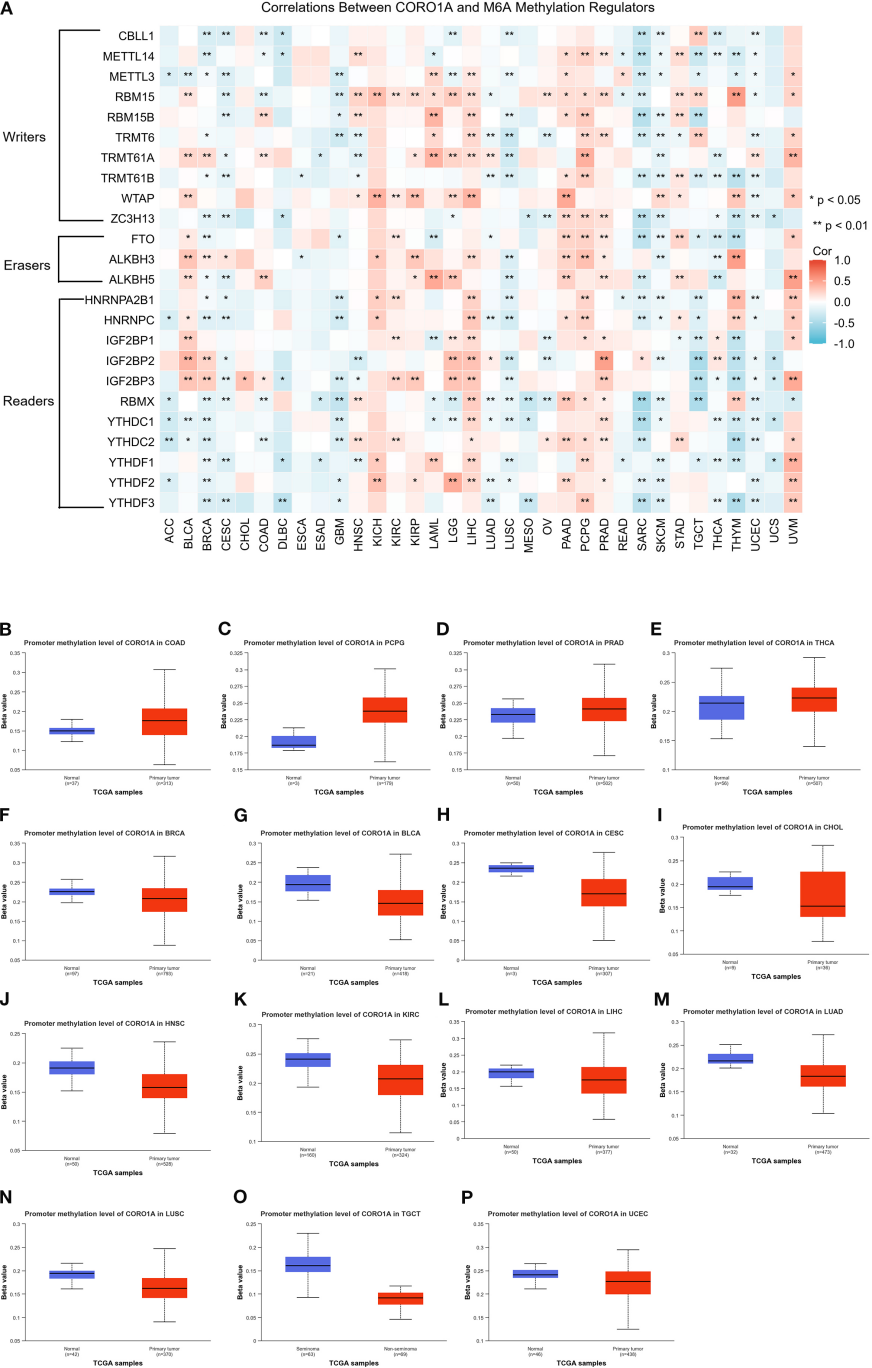
Analysis of CORO1A epigenetic methylation. **(A)** The connection between CORO1A mRNA expression and m6A methylation regulatory factors across various malignancies. Levels (beta values) of CORO1A promoter methylation in normal tissues and malignancies as analyzed by UALCAN, encompassing COAD **(B)**, PCPG **(C)**, PRAD **(D)**, THCA **(E)**, BRCA **(F)**, BLCA **(G)**, CESC **(H)**, CHOL **(I)**, HNSC **(J)**, KIRC **(K)**, LIHC **(L)**, LUAD **(M)**, LUSC **(N)**, TGCT **(O)**, and UCEC **(P)**. *p<0.05, **p<0.01.

Here, the connection between the CORO1A mRNA expression levels and the m6A methylation inhibitors was explored across a spectrum of cancers, recognizing the pivotal function that m6A methylation contributed to cancer progression. Our study led to the discovery of 24 pivotal regulators of m6A methylation. Comprising 3 erasers (FTO, ALKBH3/5), 10 writers (CBLL1, ZC3H13, METTL3/14, RBM15/15B, TRMT61A/B, TRMT6, WTAP), and 11 readers (HNRNPA2B1, IGF2BP1/2/3, RBMX, YTHDF1/2/3, YTHDC1/2, HNRNPC). Heat map analysis revealed that CORO1A expression in LIHC, PCPG, and UVM is associated with specific m6A methylation regulators (**Figure 6A**). Additionally, we compared CORO1A promoter methylation levels between healthy and malignant tissues deploying the UALCAN database. The study (**Figures 6B–E**) identified CORO1A promoter hypermethylation in COAD, PCPG, PRAD, and THCA, and hypomethylation in BRCA, BLCA, CESC, CHOL, HNSC, KIRC, LIHC, LUAD, LUSC, TGCT, and UCEC relative to healthy tissues (**Figures 6F–P**).

This study investigated the link between CORO1A methylation patterns and ICI using the TISDIB database. Heat maps indicated a reverse association between CORO1A methylation levels alongside the ICI for most immune cells (**Supplementary Figure S2A**). Additionally, the implication of CORO1A promoter methylation on mRNA expression and patient survival across various cancers was analyzed employing the GSCA database. A significant connection was ascertained between CORO1A promoter methylation and mRNA expression in the majority of the 33 malignancies from the TCGA database, except for DLBC and OV (**Supplementary Figure S2B**). Moreover, CORO1A promoter hypomethylation adversely affects the prognosis of patients with UVM, KIRC, or LGG (**Supplementary Figure S2C**).

In conclusion, hypomethylation of the CORO1A promoter may be linked to cancer prognosis and immune invasion.

### 3.5 Characteristics of genetic changes in CORO1A

**Figure 7:**
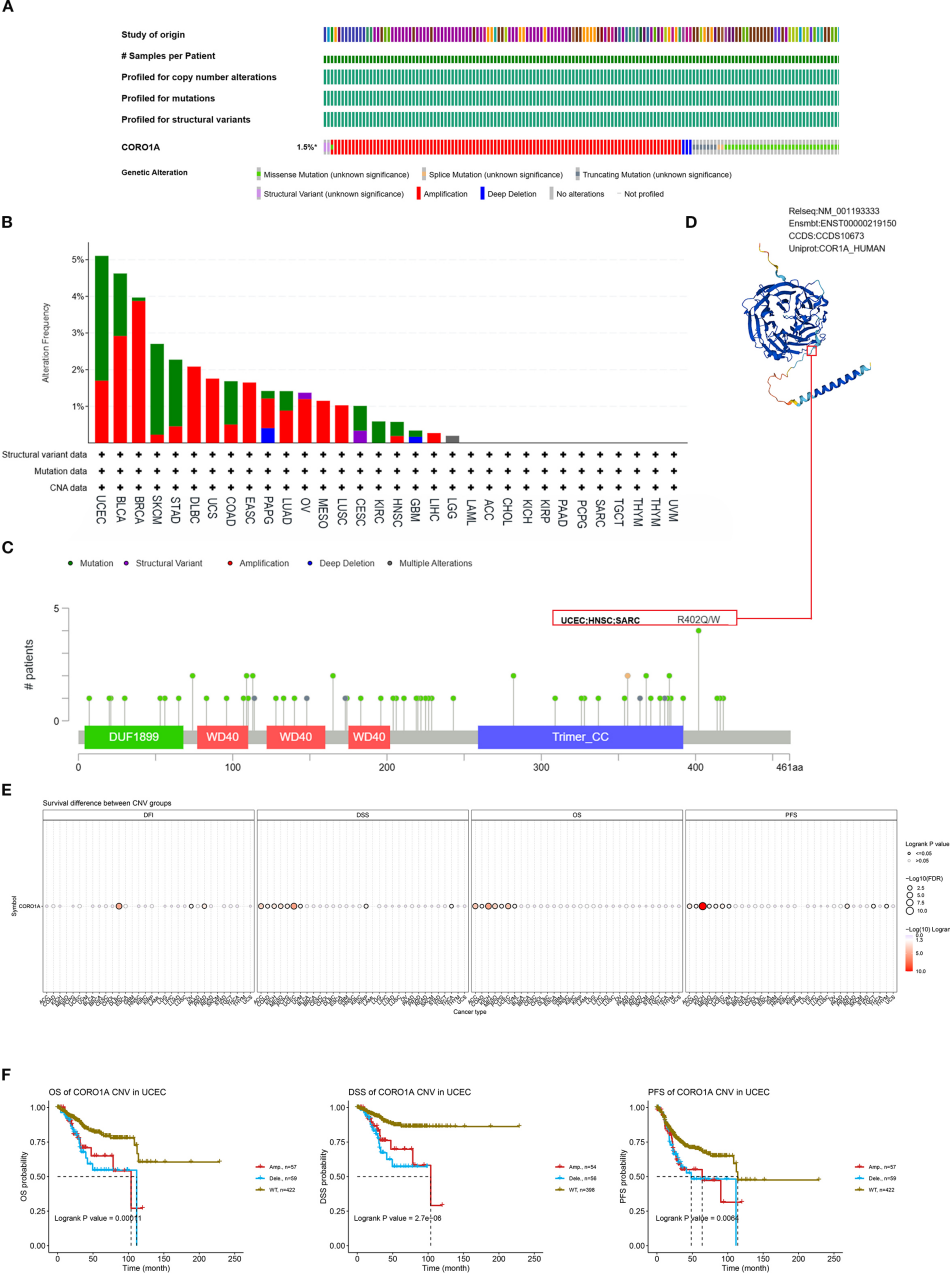
Mutated characteristics of CORO1A in various tumors. **(A)** Overview of alterations in CORO1A expression across various cancers. **(B)** Bar plot depicting the frequency and types of CORO1A alterations in various cancer types. **(C)** The landscape of CORO1A mutations concerning their location, types, number, and association with protein domains. **(D)** Certain mutations in CORO1A are displayed on the protein’s 3D structure. **(E)** The connection between CNV in CORO1A and cancer patient prognosis. **(F)** Correlation between CNV in CORO1A and prognosis in UCEC patients, including OS, DSS, and PFS.

Genetic alterations drive cancer, with some serving as potential targets for molecular therapy. Consequently, we deployed the cBioPortal database to determine the profile of CORO1A genetic variation in pan-cancer. Analysis revealed that CORO1A mutations occurred in 168 (1.5%) of the 10,967 pan-cancer samples, predominantly as missense mutations (**Figure 7A**). Missense substitutions accounted for 54.41% of all mutations, while synonymous substitutions comprised 25.29%. The SNV category was the most prevalent, with G > A mutations accounting for 40.78%, followed by C > T mutations at 27.18% (**Figure 7B**) highlights the five cancer types with the highest frequency of CORO1A mutations: BLCA, BRCA, SKCM, STAD, and UCEC. The R402Q/W mutation site is the most common for CORO1A, identified in one UCEC patient, one HNSC patient, and two SARC patients (**Figure 7C**). The CORO1A protein 3D structure is presented, highlighting the R402Q/W position (**Figure 7D**).

This study investigated the link between CORO1A mutations, CORO1A mRNA expression, and pan-cancer patient prognosis. The CNV mutations in CORO1A correlate with worse prognosis in ACC, COAD, KICH, MESO, PCPG, and UCEC patients (**Figure 7E**). Specifically, CORO1A deletion mutations in UCEC are linked to shorter OS, DSS, and PFS (**Figure 7F**). The CNV pie chart indicates that heterozygous amplifications and deletions are prevalent across most cancers. Rare homozygous amplifications are mainly observed in BRCA, DLBC, LUAD, MESO, BLCA, and UCS, while rare homozygous deletions are primarily found in LAML and TGCT (**Supplementary Figure S3A**). A negative correlation was identified between CNV occurrence in CORO1A and its mRNA expression levels in cancers like LGG, BRCA, and KIRP (**Supplementary Figure S3B**). Consequently, mutations in the CORO1A gene are prevalent in a broad range of cancers and correlate with the clinical outcomes for cancer patients.

### 3.6 CORO1A is implicated in pan-cancer immune invasion and response

**Figure 8:**
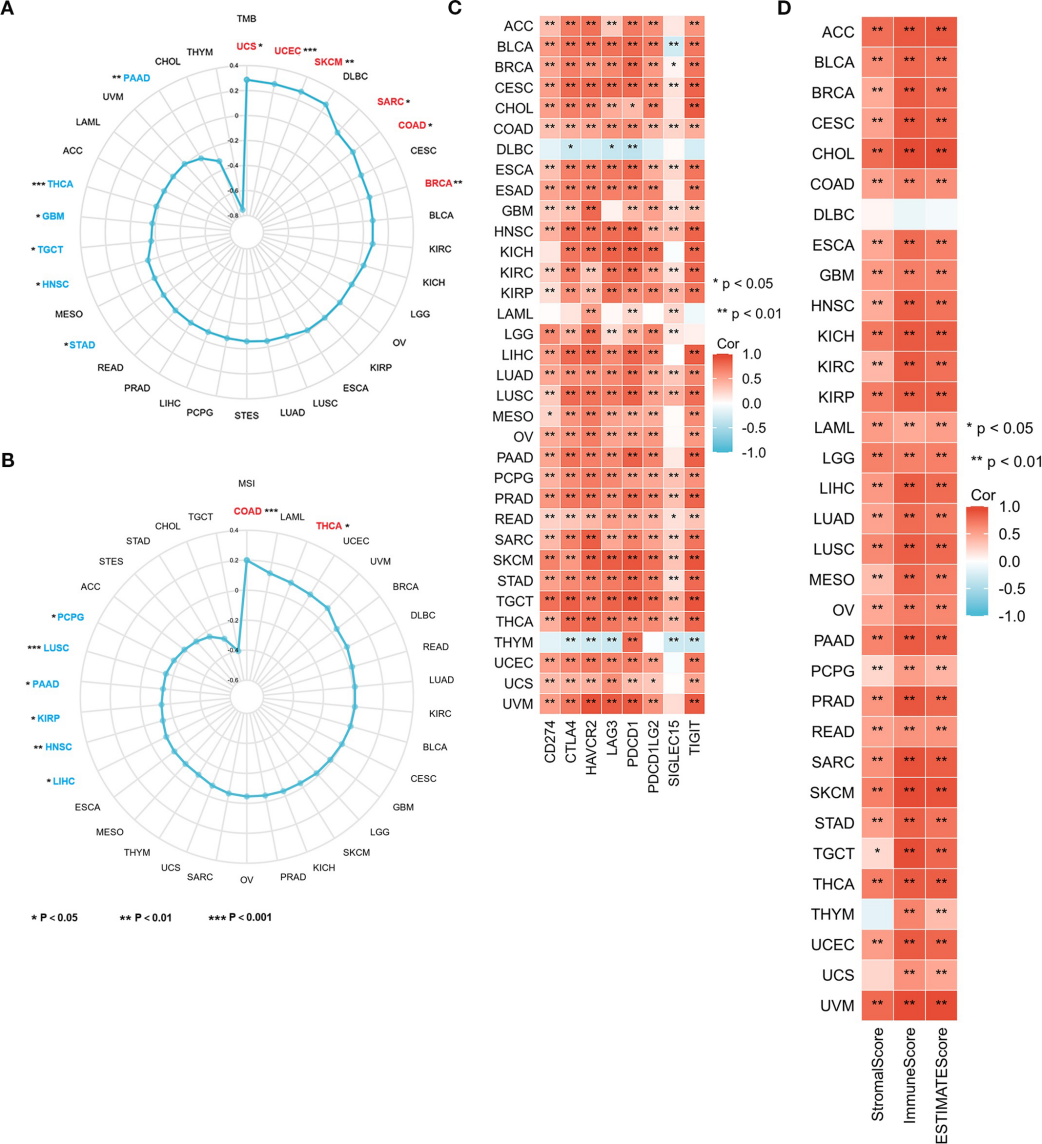
CORO1A expression is connected with TMB, MSI, TME, and immune checkpoints in 33 cancer types. Correlation between CORO1A expression and TMB **(A)**, MSI **(B)**, immune checkpoint expression **(C)**, as well as Stromal, Immune, and ESTIMATE Scores **(D)** in 33 cancers. *p<0.05, **p<0.01.

Tumor mutation burden (TMB) and Microsatellite Instability (MSI) serve as prognostic biomarkers for evaluating cancer patients’ responses to immunotherapy. This study examined the association between CORO1A mRNA levels and the parameters of TMB and MSI, revealing an inverse connection between CORO1A mRNA expression and TMB in the following cancers, with correlation coefficient (R) as follows: GBM (– 0.13), HNSC (– 0.11), PAAD (– 0.24), STAD (– 0.10), THCA (– 0.18), and TGCT (– 0.16). Conversely, a positive correlation was observed in several cancers with R values as follows: BRCA (0.09), COAD (0.13), SARC (0.14), SKCM (0.27), UCEC (0.27), and UCS (0.29) (P < 0.05; **Figure 8A**).

Additionally, CORO1A mRNA expression exhibited a positive connection with MSI in the following cancers, with R values: COAD (0.20) and THCA (0.10) and an adverse association in HNSC (-0.13), KIRP (- 0.14), LIHC (-0.11), LUSC (-0.15), PAAD (-0.15), and PCPG (-0.17) (P < 0.05; **Figure 8B**).

Our research exhibited a substantial positive link between CORO1A levels and the immune checkpoint protein expression in a range of malignancies (P < 0.05; **Figure 8C**). Moreover, we ascertained the link between CORO1A mRNA levels and tumor stromal, immune invasion, and purity scores in pan-cancer. The heat map displays CORO1A expression in 30 cancer types, encompassing ACC, BLCA, BRCA, CESC, CHOL, COAD, ESAD, GBM, HNSC, KICH, KIRC, KIRP, LAML, LGG, LIHC, LUAD, LUSC, MESO, OV, PAAD, PCPG, PRAD, READ, SARC, SKCM, STAD, TGCT, THCA, UCEC, and UVM. The mRNA expression level manifests a positive connection with both tumor stromal and immune invasion scores (P < 0.05; **Figure 8D**). Our analysis assessed the correlation between the CORO1A expression levels and several immune-linked genes, encompassing 43 genes associated with immune activation, 22 genes linked to immune suppression, 21 major MHC genes, 41 chemokines, and 18 chemokine receptor genes. Additionally, we exhibited that CORO1A expression is positively linked to these immune-related genes across a variety of cancers (**Supplementary Figures S5A–E**). A majority of cancers show significant relationships between CORO1A expression and immune scores, immune checkpoints, and gene expression related to immunity.

**Figure 9:**
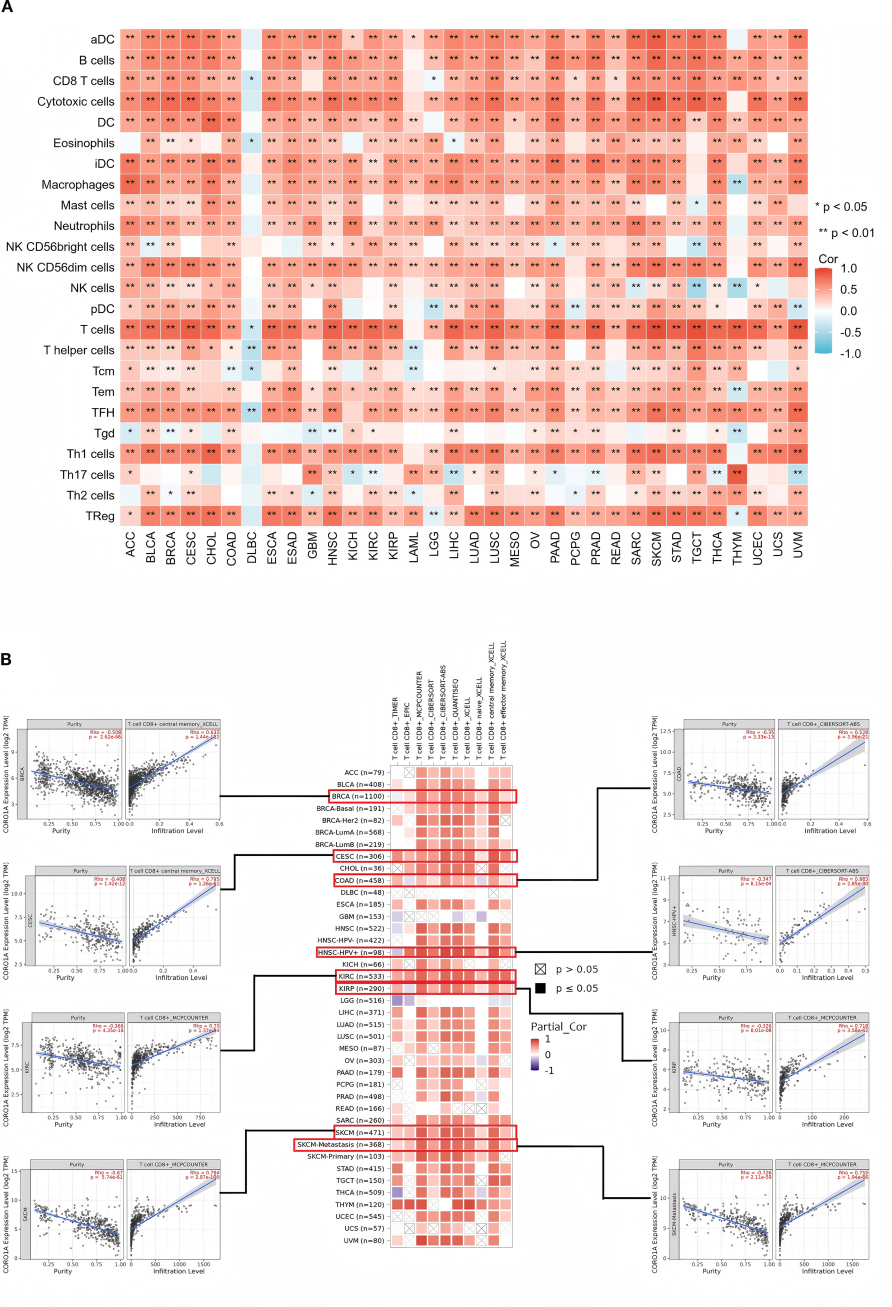
Connections between ICI levels and CORO1A expression in pan-cancer. **(A)** The link between CORO1A expression and ICI assessed by the ssGSEA methodology. **(B)** Correlation analysis between CORO1A expression and the CD8 T cells infiltration with the Timer2.0 database, including scatter graphs for BRCA, CESC, COAD, KIRC, KIRP, and SKCM. Immune cell infiltration, ICI. *p<0.05, **p<0.01.

Tumor-infiltrating immune cells (TIICs) are crucial to the tumor microenvironment (TME) and impact tumor aggressiveness. Using the ssGSEA method, we ascertained the correlation between the CORO1A mRNA expression levels and the presence of 24 TIICs. Our heatmap analysis exhibited the CORO1A mRNA levels possessed a significant positive correlation with most TIICs infiltration in a broad spectrum of 31 different cancer types, encompassing ACC, BLCA, BRCA, CESC, CHOL, COAD, ESCA, ESAD, GBM, HNSC, KICH, KIRC, KIRP, LAML, LGG, LIHC, LUAD, LUSC, OV, PAAD, PCPG, PRAD, READ, SARC, SKCM, STAD, TGCT, THCA, UCEC, UCS, and UVM (P < 0.05; **Figure 9A**). Additionally, we utilized EPIC, MCP-COUNTER, and TIDE algorithms within the Timer 2.0 database to analyze the link between CORO1A mRNA expression and TIIC infiltration, revealing a positive association between CORO1A mRNA levels and CD8+T cell infiltration across multiple cancer types. Significant correlations were identified in six cancer types, with R values: BRCA (0.633), CESC (0.795), COAD (0.528), KIRC (0.75), KIRP (0.718), and SKCM (0.794) (P < 0.05; **Figure 9B**). Additionally, CORO1A expression was notably linked to T cell and macrophage invasion across various cancers (**Supplementary Figure S5A**).

### 3.7 Functional enrichment analysis of CORO1A

**Figure 10:**
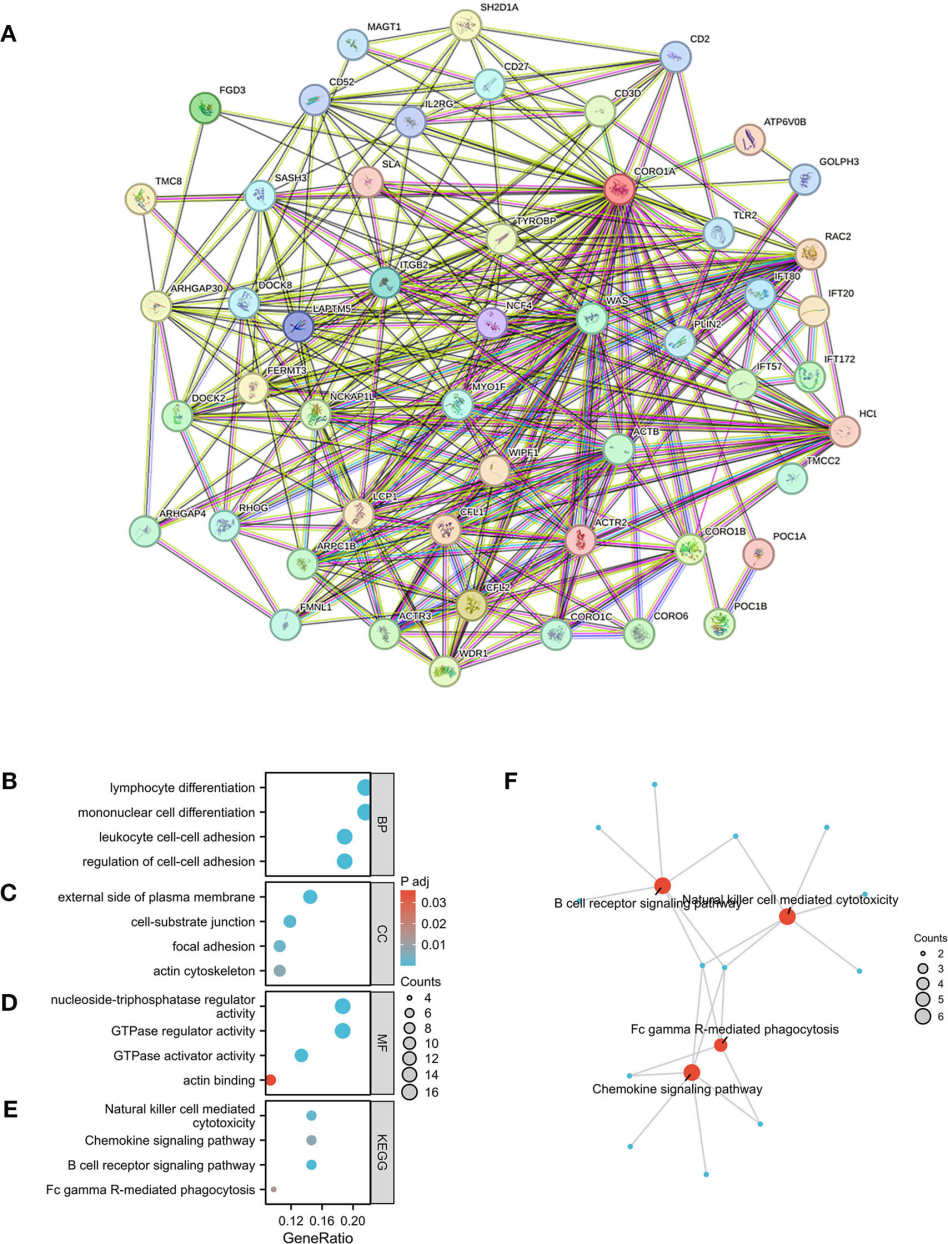
CORO1A-linked genes, interacting proteins, and functional enrichment analysis. **(A)** PPI Network for CORO1A. **(B)**, cellular component **(C)**, molecular function **(D)**, and KEGG pathway **(E)**. **(F)** Visual network of KEGG analyses.

Herein, we conducted an enrichment analysis of genes co-expressed with CORO1A to understand its possible molecular pathways in tumorigenesis and development. Based on the GEPIA2 database, the top 100 genes that were co-expressed with CORO1A were determined (**Supplementary Table S2**). A PPI network, constructed using 50 CORO1A-binding proteins from the STRING database, manifested 51 nodes, 320 edges, an average node degree of 12.5 (P < 0.001, **Figure 10A**). The first 100 genes co-expressed with CORO1A were analyzed for functional enrichment, revealing 16 KEGG pathways and 311 GO categories, comprising 275, we identified 28 cellular components (CC), 8 molecular functions (MF), and biological processes (BP) (**Supplementary Table S3**). Four cancer-related items were randomly selected from each GO category. The BP analysis highlighted roles in lymphocyte differentiation, unicellular differentiation, leukocyte-cell adhesion, and cell adhesion (**Figure 10B**). Regarding CC, these genes were enriched in a predominant localization on the outer plasma membrane, cell-substrate junction, focal adhesion, and actin cytoskeleton (**Figure 10C**). Moreover, MF includes nucleoside triphosphatase and GTPase regulatory activities, GTPase activator activity, and actin-binding (**Figure 10D**). The KEGG pathway analysis revealed pathways including cytotoxic actions by NK cells chemokine signaling, B-cell receptor-mediated signaling, and FcγR-mediated phagocytosis (**Figure 10E**). Visualization indicated that the FcγR-mediated phagocytosis pathway exhibited the greatest gene overlap, suggesting its potential involvement in mediating CORO1A (**Figure 10F**).

**Figure 11:**
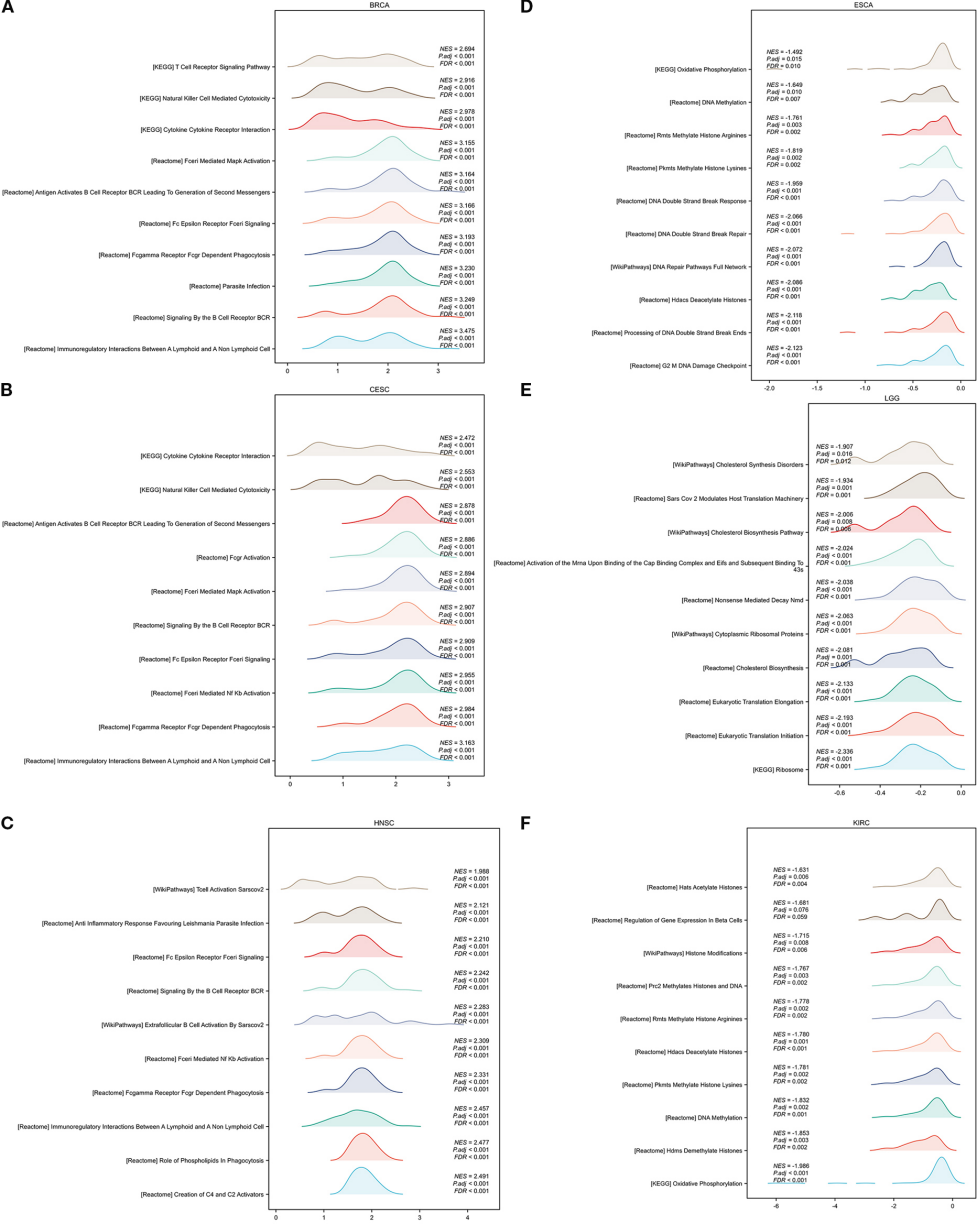
GSEA functional enrichment analysis of CORO1A in 6 cancers. The top 10 reaction pathways in BRCA **(A)**, CESC **(B)**, and HNSC **(C)** were positively connected with CORO1A expression. The first 10 reaction pathways in ESCA **(D)**, LGG **(E)**, and KIRC **(F)** negatively associated with CORO1A expression.

In addition, we conducted GSEA analysis using the respondome pathway database to investigate CORO1A’s potential mechanism of action in pan-cancer. Our analysis included six cancer types: BRCA, CESC, and HNSC showed a positive prognosis with CORO1A expression, while ESCA, LGG, and KIRC exhibited a negative prognosis. The GSEA findings revealed that in cancer types with a favorable prognosis linked to CORO1A, genes exhibiting a positive connection with CORO1A were mostly abundant in immune-linked pathways (**Figures 11A–F**). Conversely, in cancer subtypes characterized by a poor prognosis, there is an enrichment of genes that exhibit a negative association with CORO1A, particularly in pathways related to immune responses. These pathways encompass interactions that modulate immunity between lymphoid and non-lymphoid cells, the phagocytic process mediated by Fcγ receptors, and the cytotoxic actions of NK cells. In KIRC, genes that show a negative correlation with CORO1A are predominantly engaged in DNA methylation, modifications to proteins after translation, and the cell cycle regulation, which includes the deacetylation of histones, their methylation, and the control points within the cell cycle (**Figure 11F**).

In conclusion, CORO1A significantly impacts cancer by modulating immune-related pathways.

### 3.8 The CORO1A expression in BRCA tissues

**Figure 12:**
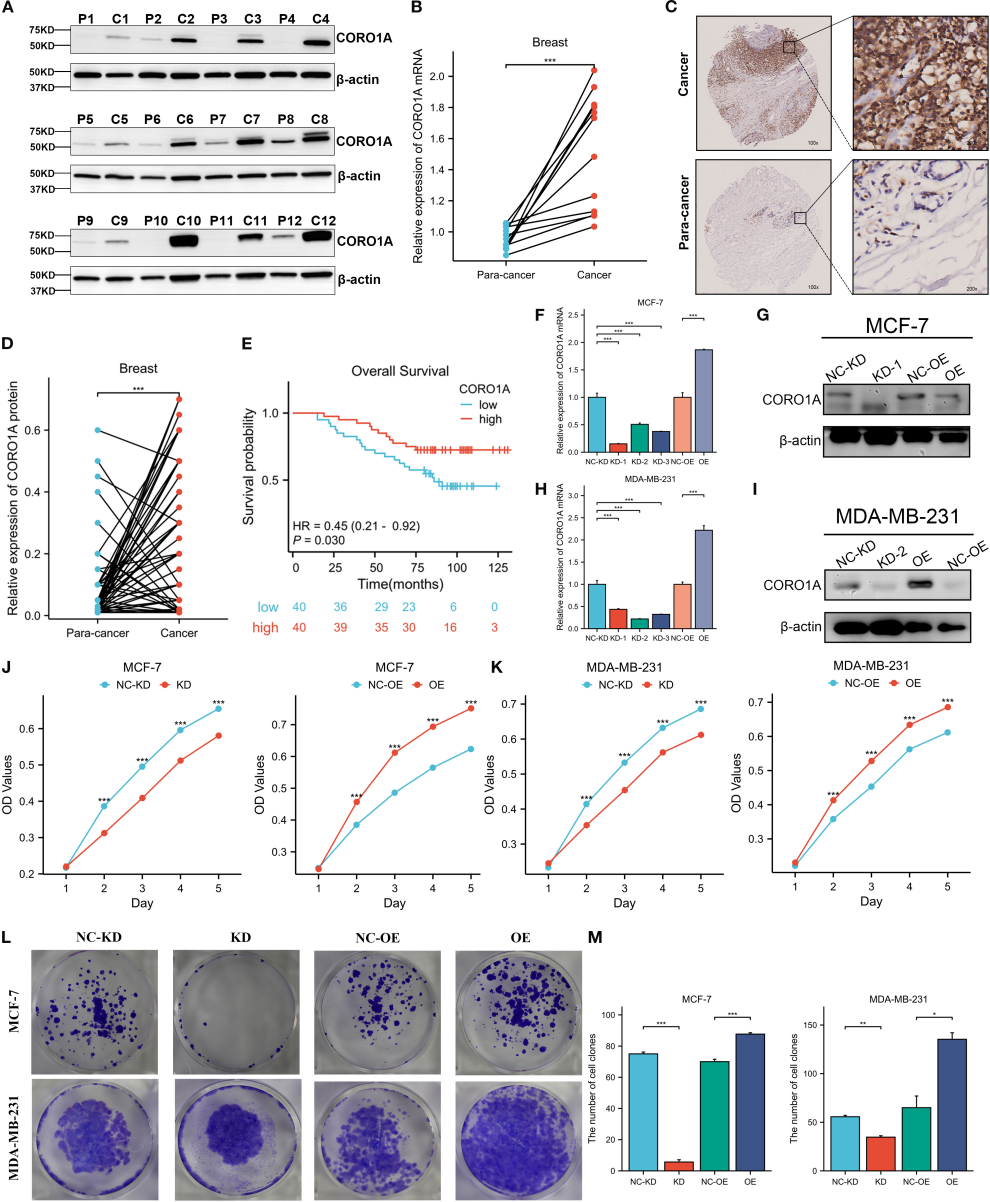
CORO1A is highly expressed in BRCA tissues and promotes the proliferation of breast cancer cells. **(A)**Western blot was deployed to confirm the CORO1A expression in cancer and adjacent tissues from 12 BRCA patients. **(B)** CORO1A mRNA expression in cancer and adjacent tissues of 12 patients (p < 0.001) **(C)** Illustrative images of immunohistochemical staining of BRCA tissues and corresponding paracancerous tissues using tissue microarray chip (n=80); **(D)** Tissue microarray chip (n=80) to detect the CORO1Ad expression in BRCA and adjacent tissues (p < 0.001); **(E)** The OS curve of high (n = 40) and low (n = 40) CORO1A expression patients by Kaplan-Meier. Breast cancer, BRCA; **(F)** The efficiency of knockdown and overexpression of CORO1A in breast cancer cell line MCF-7 was verified by RT-qPCR. **(G)** WB experiments were used to verify the efficiency of knockdown and overexpression of CORO1A in breast cancer cell line MCF-7. **(H)** The efficiency of knockdown and overexpression of CORO1A in breast cancer cell line MDA-MB-231 was verified by RT-qPCR. **(I)** WB experiments were used to verify the efficiency of knockdown and overexpression of CORO1A in breast cancer cell line MDA-MB-231. **(J, K)** CCK-8 assay was used to verify the proliferation ability of breast cancer cell lines (p < 0.001). **(L)** The proliferation ability of breast cancer cell lines was verified by colony formation assay. **(M)** The number of knockdown and overexpression clones of CORO1A in breast cancer cell lines. (p < 0.05). *p<0.05, **p<0.01, ***p<0.001.

**Figure 13:**
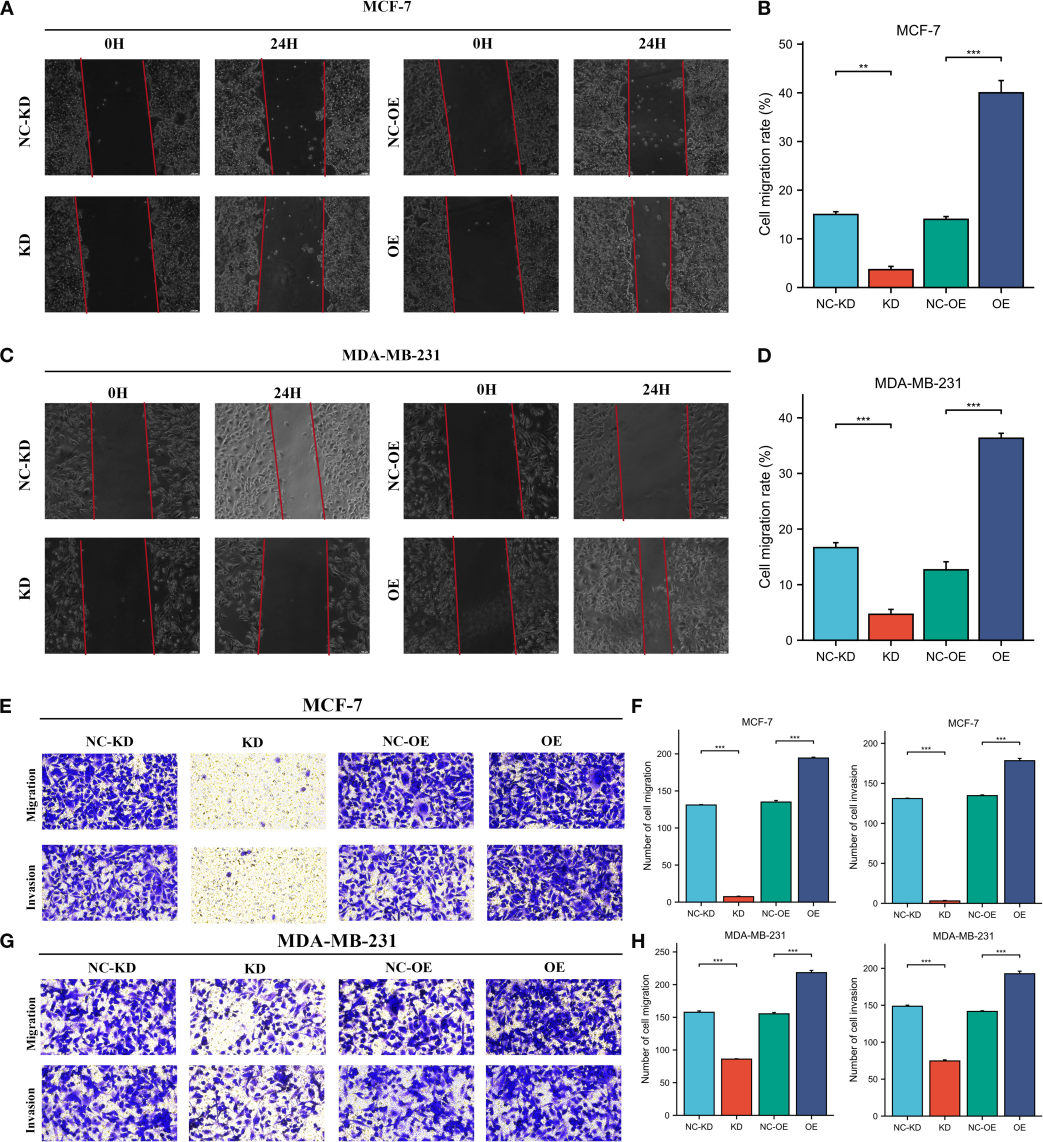
High expression of CORO1A can enhance the migration and invasion abilities of breast cancer cell lines. **(A, B)** Cell scratch assay was used to verify the cell migration ability of MCF-7 cells after knockdown and overexpression of CORO1A (p < 0.001). **(C, D)** Cell scratch assay was used to verify the cell migration ability of MDA-MB-231 cells after knockdown and overexpression of CORO1A (p < 0.0001). **(E, F)** Transwell assay was used to verify the changes of cell invasion and migration ability after knockdown and overexpression of CORO1A in MCF-7 cells (p < 0.0001). **(G, H)** ranswell assay was used to verify the changes of cell invasion and migration ability after knockdown and overexpression of CORO1A in MDA-MB-231 cells (p < 0.0001). ***p<0.001.

Herein, we collected 12 BRCA specimens and their adjacent normal tissues to validate CORO1A expression from the First Affiliated Hospital of Xinjiang Medical University. Western Blot analysis manifested that CORO1A expression significantly raised in BRCA tissues, unlike adjacent normal tissues (P < 0.001, **Figures 12A, C**; **Supplementary Figure S6**). Moreover, we assessed the clinical relevance of CORO1A expression in BRCA by conducting immunohistochemical staining on a microarray of 80 paired BRCA and adjacent tissue samples. The findings revealed a significantly elevated level of CORO1A expression within malignant tissues (P < 0.001, **Figures 12B, D**). Moreover, KM survival analysis of the clinical data from these patients indicated that greater CORO1A expression is linked to a better prognosis (**Figure 12E**). Overall, CORO1A is significantly overexpressed in BRCA tissues and is linked to improved patient outcomes. To explore the impact of different expression levels of CORO1A on the malignant phenotype of breast cancer, breast cancer cell lines MCF-7 and MDA-MB-231 were established with CORO1A knockdown and overexpression, and validated by RT-qPCR and Western Blot (**Figures 12F–I**). CCK-8 and colony formation assay results indicate that CORO1A overexpression promotes the proliferation of breast cancer cell lines, while knockdown has the opposite effect (**Figures 12J–M**). Cell scratch assay (**Figures 13A–D**) and Transwell assay (**Figures 13E–H**) results show that CORO1A overexpression significantly enhances the migration and invasion capabilities of breast cancer cell lines, whereas knockdown yielded opposite results. In summary, experimental results demonstrate that CORO1A is highly expressed in breast cancer and promotes the proliferation, migration, and invasion capabilities of breast cancer.

## 4 Conclusion

Cancer is a serious disease caused by malignant proliferation and abnormal multiplication of cells, which is highly aggressive and metastatic and poses a significant threat to human health^[2]^. Its etiology is complex, often resulting from prolonged exposure to physical, chemical, and viral factors (26). Treatment options include surgery, chemoradiotherapy, and targeted therapy, with the latter being essential for precisely targeting specific molecules in cancer cells to inhibit their growth and spread (27). Despite the identification of some therapeutic markers, many cancers still lack effective targets (28). Consequently, discovering potent molecular markers with multiple targets could offer novel diagnostic and therapeutic strategies for cancers without effective clinical targets.

The CORO1A, a highly exceptionally conserved Coronins family of intracellular actin-binding proteins, is essential for phagocytosis, cell migration, and cytoskeletal reorganization. It is essential for immune response regulation and acts as a key pro-inflammatory cytokine. The CORO1A may affect cell signaling pathways and is considered a possible target for treating cancer (8, 29–31).

First, we aimed to analyze the CORO1A gene expression in pan-cancer tissues to evaluate its role in cancer. By using the TCGA database to collect data from different pan-cancer tissues in more than 30 cancers, we found overexpression of the CORO1A gene and a significant correlation between CORO1A levels and immune subsets. Among them, the expression of CORO1A reached the highest level in the C2 immune subtype, which may be related to its role as a pro-inflammatory cytokine in regulating immune response.

Subsequently, we further investigated the CORO1A impact on the pan-cancer prognosis. Among various cancer types, we found that CORO1A exhibited differences in prognosis. Our data suggest that high CORO1A expression manifested the connection with better prognosis in cancer types such as BRCA, CESC, HNSC, SKCM, UCEC, and LUAD, among others. This may suggest that CORO1A significantly contributed to the inhibition of cancer progression in the progression of these cancers, which is aligned with prior reports: Ding et al. (32) have identified seven key genes in HNSC by multivariate COX regression analysis, with high CORO1A expression connected with better prognosis. This discovery indicates that CORO1A may function as a significant predictive indicator in HNSC, with elevated expression potentially predicting a favorable outcome for patients. Meanwhile, Zhou et al. (33) have revealed that high CORO1A levels were connected with longer PFS and OS in LUAD. This indicates that CORO1A may serve as a beneficial prognostic marker in LUAD, with elevated expression potentially enhancing patient survival. Furthermore, in SKCM, elevated levels of CORO1A expression have been correlated with improved survival results (34). However, in cancer types such as ESCA, KIRC, and LGG, its high expression levels may indicate that the tumor is more aggressive and metastasis, which in turn leads to a worse prognostic outcome. Our results are aligned with current reports: Wang et al. (35) have found that high CORO1A expression was negatively linked to patient survival in esophageal squamous cell carcinoma (ESCA), i.e., the higher the level of CORO1A expression in ESCA, the shorter the survival of patients. Furthermore, this supports the importance of CORO1A in cancer prognosis and suggests that it may exhibit various prognostic functions in different cancer types. Subsequently, we performed a thorough study of the diagnostic efficacy of CORO1A in pan-cancer and identified that it exhibited the most favorable diagnostic outcomes in three cancers: SKCM, sarcoma (SARC), and KIRC. Especially in SKCM, the area under the curve is as high as 0.98, showing extremely high diagnostic accuracy. Therefore, we speculate that CORO1A may be a promising molecular diagnostic marker in SKCM. In addition, some studies have shown that CORO1A has shown good diagnostic value in osteosarcoma, which is consistent with our conclusions (36). In addition, the use of CORO1A in tumor diagnosis is not limited to specific cancers, and it has been shown to be of high value in the diagnosis of THYM (13). Similarly, Hu et al. (37) have manifested significant elevation CORO1A expression levels in renal fibrosis specimens and showed good diagnostic efficacy, suggesting that CORO1A may also have some potential in the diagnosis of renal fibrosis. In summary, CORO1A is predicted to be a novel tumor diagnosis and prognostic marker.

Second, we analyzed the epigenetic modification of CORO1A and found no significant association between CORO1A and the molecule that regulates M6A methylation. However, in most cancers, the degree of methylation of the CORO1A gene promoter region is significantly mitigated, unlike that of normal tissues, which means that the protein encoded by CORO1A may be present at higher levels in cells, which in turn affects the biological behavior of cells. Among the two cancers, KIRC and LUAD, the promoter hypomethylation status of CORO1A may help promote cancer progression. However, due to the incomplete understanding of the methylation regulation mechanism of the promoter of the CORO1A gene, the conclusion that the hypomethylation status of the promoter may promote the progression of KIRC and LUAD cancers has not been experimentally confirmed. Notably, in PRAD, we observed a significantly greater methylation level in the CORO1A gene promoter region than in normal tissues. This finding suggests that in PRAD, hypermethylation of the CORO1A promoter region may lead to silencing of the expression of this gene, thereby improving the tumor cells’ capability to proliferate, migrate, and invade, increasing the degree of malignancy of tumors and ultimately contributing to a worse prognosis. Demethylation therapy against PRAD may help restore CORO1A expression, thereby inhibiting the malignant behavior of tumor cells. Consequently, CORO1A is anticipated to serve as a novel target for treating PRAD. Furthermore, we discovered that the methylation level of the CORO1A promoter exhibited an inverse association with most of the ICI levels. Consequently, CORO1A may be classified as an immune-related gene, and in tumor cells, elevated promoter methylation levels result in the down-regulation of CORO1A gene expression, which may weaken tumor recognition and ICI.

Our analysis of CORO1A genetic variations revealed that missense mutations are predominant, with R402Q/W being the most frequent mutation site. The CORO1A gene variant is significantly associated with UCEC prognosis, possibly due to missense mutations causing gene deletion, resulting in down-regulation and adverse outcomes in UCEC patients. Khoreva et al. (38) have discovered a homozygous missense mutation in the CORO1A gene, causing a conserved amino acid substitution in the β-helix region and resulting in the absence of CORO1A protein expression in patient cells. Studies suggest that CORO1A gene mutations contribute to immunodeficiency in T, B, and NK cells, heightening the risk of severe viral infections and possibly promoting cancer development (39–41). Our findings indicate an inverse relationship between CNV and CORO1A gene expression, implying that elevated CORO1A expression is susceptible to deletion, resulting in its down-regulation and potentially contributing to a poorer prognosis. Giannuzzi et al. (25) identified a significant heterozygous deletion on chromosome 16’s short arm, encompassing the CORO1A gene, and discovered a novel hemizygous CORO1A variant C > T, aligning with our findings.

Recent advancements in tumor immunotherapy have established adoptive T cell therapy (ACT) and immune checkpoint inhibitor therapy as primary treatment methods (42). Higher levels of TMB and MSI are important indicators of improved patient outcomes in immune checkpoint inhibitor therapy (15, 43). Our analysis of 33 cancer types identified a strong expression of CORO1A in COAD, positively correlating with TMB and MSI. Moreover, we propose that high CORO1A expression contributes to elevated TMB and MSI in COAD, potentially enhancing patient response to immune checkpoint inhibitor treatment. The study identified a favorable association between CORO1A expression and many immune checkpoints, suggesting that elevated CORO1A levels may enhance immune checkpoint function, supporting immune homeostasis and antitumor immunity. This indicates that high CORO1A expression could be a significant target for tumor therapy. Additionally, CORO1A expression in tumor tissues is positively linked to the tumor stromal fraction and immune invasion score, implying increased stromal components and enhanced immune infiltration, leading to greater immune cell presence and activity in tumor tissues. Therefore, CORO1A-dependent therapy may be a potential treatment for certain cancers.

The TME is integral to tumor initiation, progression, and treatment, influencing these processes through complex interactions with epithelial cells (44). The prognosis of many tumors is significantly affected by ICI (45). Research indicates that tumor progression may be linked to cancer cells’ capacity to evade immune surveillance (46). Analyzing ICI in the TME can enhance our comprehension of CORO1A’s function in cancer. Moreover, we ascertained CORO1A as a key regulator of tumor immune function, showing significantly elevated expression in immune cells encompassing CD8+ and CD4+ T cells, B and NK cells, and neutrophils. These findings highlight CORO1A’s importance in immune cell function. Previous research indicates that the CORO1A gene has diverse biological roles, with its NH2 terminus being essential for T cell survival and antiviral immune responses (47). The absence of CORO1A may hinder T cell development, homeostasis, and function due to impaired T cell receptor (TCR) signaling (48). Niu et al. (49) have demonstrated a strong link between a core set of genes, including CORO1A, and immune cells, suggesting that they are significantly involved in enhancing immune system activity. Zhao et al.^[14]^ have concluded that in the C4 immune subtype of SKCM, CORO1A may exhibit higher sensitivity to drug therapy through NK cell and T cell-mediated immunopositivity regulatory mechanisms. Arandjelovic et al. (50) found that the lack of CORO1A may result in abnormal CD4+ T cell functionality. Latour et al. (51) found that anti-CORO1A monoclonal antibodies effectively treat B-cell malignancies and T-cell-induced autoinflammatory diseases. This indicates that CORO1A expression significantly influences immune response balance. Consequently, we suggest that CORO1A enhances the antitumor immune response by promoting ICI. Abnormal CORO1A expression leads to immune deficiencies, underscoring its crucial role in immune homeostasis.

Finally, we performed data analysis on the CORO1A-mediated molecular pathway in pan-cancer. Through functional enrichment analysis, it was found that the molecular pathways of CORO1A contribute to several cellular functions and BP, encompassing the regulation of cell adhesion and its binding to actin and the promotion of the enrichment of CC in the actin cytoskeleton.CORO1A, as an actin-binding protein, is able to bind to actin and thus participate in the remodeling of the cytoskeleton, a process that is crucial for maintaining the cytoskeleton stability and function (8, 29). It is also involved in cytoskeleton-related signaling pathways, thereby regulating changes in the cytoskeleton, thereby regulating cell division, differentiation, and migration^[2,3]^. Ojeda et al. (52) have suggested that CORO1A may affect the cytoskeleton and signaling by regulating the Rac1 GTPase and myosin II-dependent signaling pathways. Castro et al. (53)suggested that CORO1A is a key binding protein of F-actin that regulates the dynamics of the cytoskeleton in a variety of microenvironments. Hölttä-Vuori et al. (23) suggested that CORO1A negatively regulates lysosomal delivery, modification of lipoprotein degradation, and cholesterol deposition in macrophages by controlling the binding of actin to endocytic organelles. Then, through KEGG pathway analysis, we found that the following four pathways were highly correlated with CORO1A: NK cell-mediated cytotoxicity, chemokine, B-cell receptor, and Fcγ R-mediated phagocytosis signaling pathway, the most common of which was Fcγ R-mediated phagocytosis. It has been reported that Fcγ R-mediated phagocytosis is mainly by controlling the phagocytosis of immune cells, encompassing macrophages, dendritic cells, and NK cells, and engulfing and eliminating tumor cells (54). This process is consistent with earlier studies demonstrating that CORO1A is significantly involved in Ca2+ signaling within macrophages and contributes to lymphocyte migration (11, 55). It has been reported in the literature that CORO1A participates in vesicular transport among NK cells, functions as a phagocytic lipoprotein, and is intricately associated with the development of lytic immune synapses (56). Using GSEA, we discovered that CORO1A expression is adversely linked to prognosis and immune function in some malignancies, while it is favorably connected with immune activity and prognosis in others. Despite its varied prognostic impact, CORO1A consistently influences immune response, enhancing cancer prognosis in pan-cancer cases. In KIRC, CORO1A expression is positively connected with prognosis and adversely with cell cycle processes and DNA methylation, aligning with our findings on its impacts on proliferation, invasion, and migration.

In addition, CORO1A also plays other important roles in molecular pathways. Pick et al. (57) have used co-immunoprecipitation and mass spectrometry to find that the transport of CORO1A to neutrophils was mainly mediated by b2 integrin, and its adhesion was controlled by LFA-1. It has been found that cyclic adenosine monophosphate element-binding protein-H can inhibit the CORO1A expression, a key gene for autophagosome-lysosomal fusion, through transcriptional regulation and overexpression of CORO1A can suppress the secretion of autophagy and inflammatory factors, suggesting that CORO1A may represent a novel target of cyclic adenosine monophosphate response element-binding protein H (58). Qiao et al. (59) have found that CORO1A, a downstream molecule of Wnt5a, had a significant suppressive impact on AM-1 cells. To conclude, CORO1A is crucial in tumor development and treatment approaches, especially in tumor immune surveillance, and as a potential target for targeted therapy.

To conclude, we comprehensively investigated the expression, prognostic significance, diagnostic factors, epigenetic traits, methylation patterns, immune associations, and enrichment analysis of CORO1A across various cancers. The results show that CORO1A shows promise as a potential predictive biomarker and a prospective molecular therapeutic target for malignant cancers. However, since the current research is mainly based on bioinformatics analysis and the correlation characteristics between the data, there are certain limitations in the scope and depth of the research, so it is still necessary to further confirm its clinical application value through extensive experimental verification and deeply explore its internal mechanism.

## Data Availability

The original contributions presented in the study are included in the article/**Supplementary Material**. Further inquiries can be directed to the corresponding author.
