# Supplementary material for: CORO1A: a pan-cancer prognosis, diagnostic and immune biomarker based on breast cancer validation
Source: Front Oncol. 2025 Oct 6;15:1670526. doi: 10.3389/fonc.2025.1670526 (PMC12535887; doi:10.3389/fonc.2025.1670526)
Supplement: Supplementary file 1 [file DataSheet1.pdf]

Supplementary materials for

A Comprehensive Assessment of CORO1A's Role as a Prognostic, Diagnostic, and Immune Biomarker in Multiple Cancer Types

Dilraba Elihamu[1†], Yongxiang Li[1†], Yiyang Wang[1†], Haiyan Cui[2], Yiting Xing[1], Haohao Peng[1], Dilimulati Ismtula[1], Chenming Guo[1*]

1 Department of Breast Surgery, Center of Digestive and Vascular Surgery, The First Affiliated Hospital of Xinjiang Medical University, Urumqi, China

2 Bayingolin Mongolian Autonomous Prefecture People's Hospital, Korla City, Xinjiang Uygur Autonomous Region, 841000, China

*Corresponding author.

† These authors contributed equally to this work and shared the first authorship.

*Correspondence: Chenming Guo, gcm_xjmu@yeah.net.

Supplementary Figures.

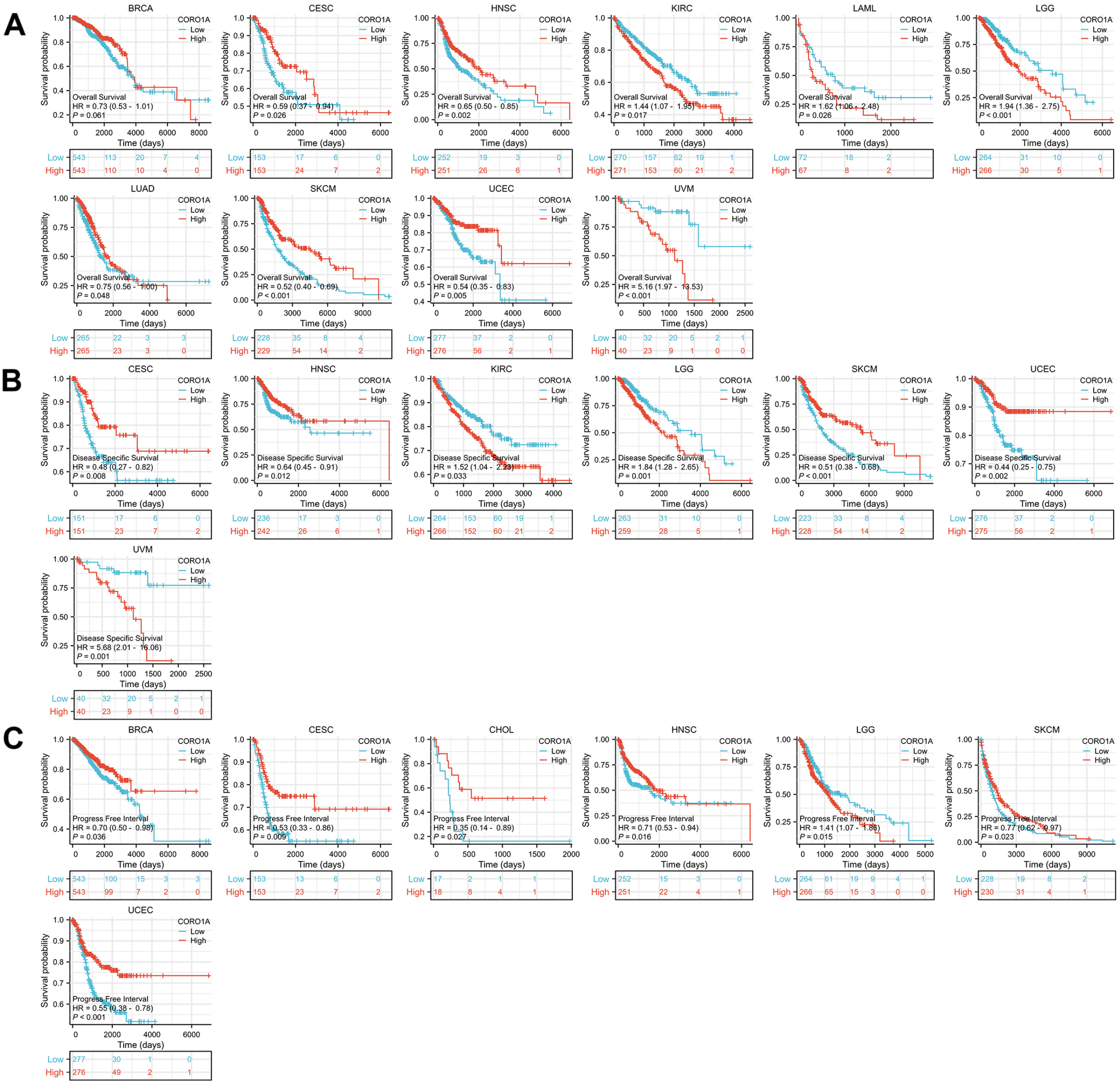

Fig.S1 The KM curve shows in detail the connection between CORO1A expression and prognosis in cancer patients. **(A)** OS in BRCA, CESC, HNSC, KIRC, LAML, LGG, LUAD, SKCM, UCEC, and UVM. **(B)** DSS in CESC, HNSC, KIRC, LGG, SKCM, UCEC, and UVM. **(C)** PFS in BRCA, CESC, CHOL, HNSC, LGG, SKCM, and UVM.

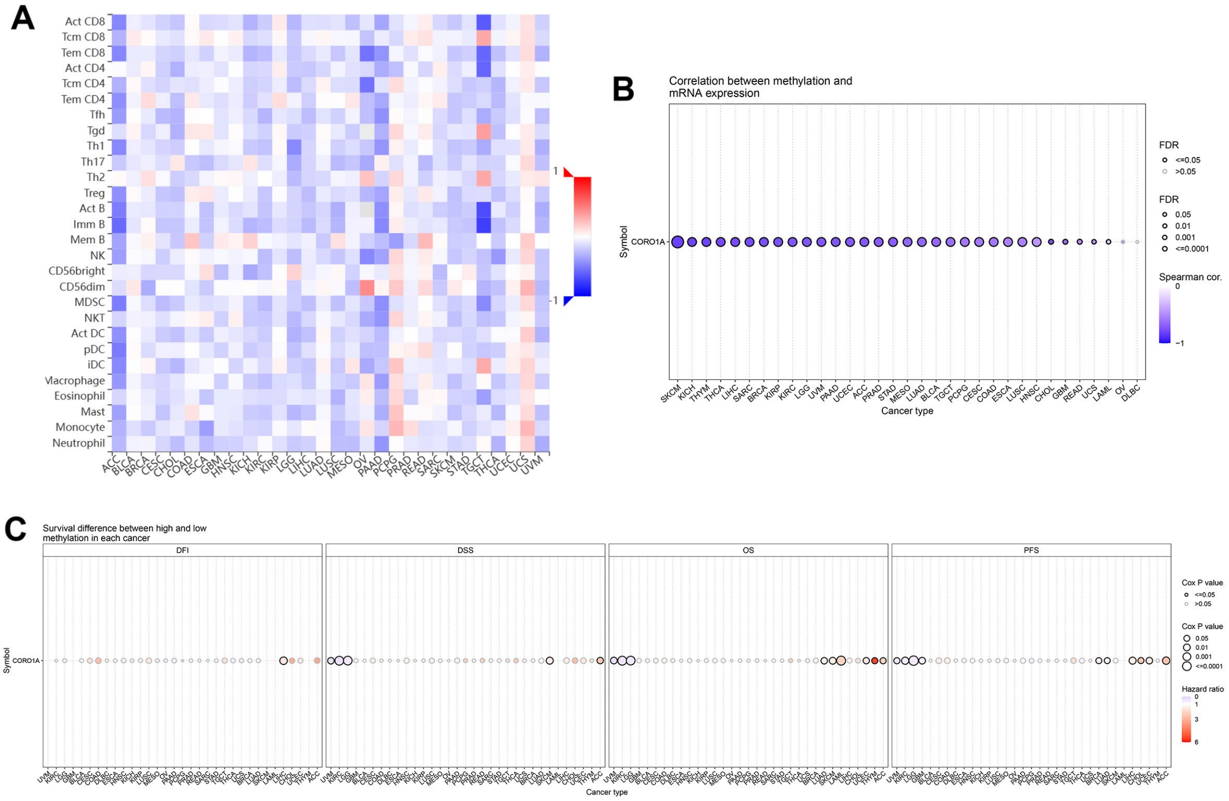

Fig.S2 Analysis of CORO1A epigenetic methylation. **(A)** Association between CORO1A methylation levels and immune cell invasion in pan-carcinoma based on TISIDB database. **(B)** Relationship between CORO1A methylation level and CORO1A mRNA expression. **(C)** Effect of CORO1A methylation levels on prognosis in cancer patients.

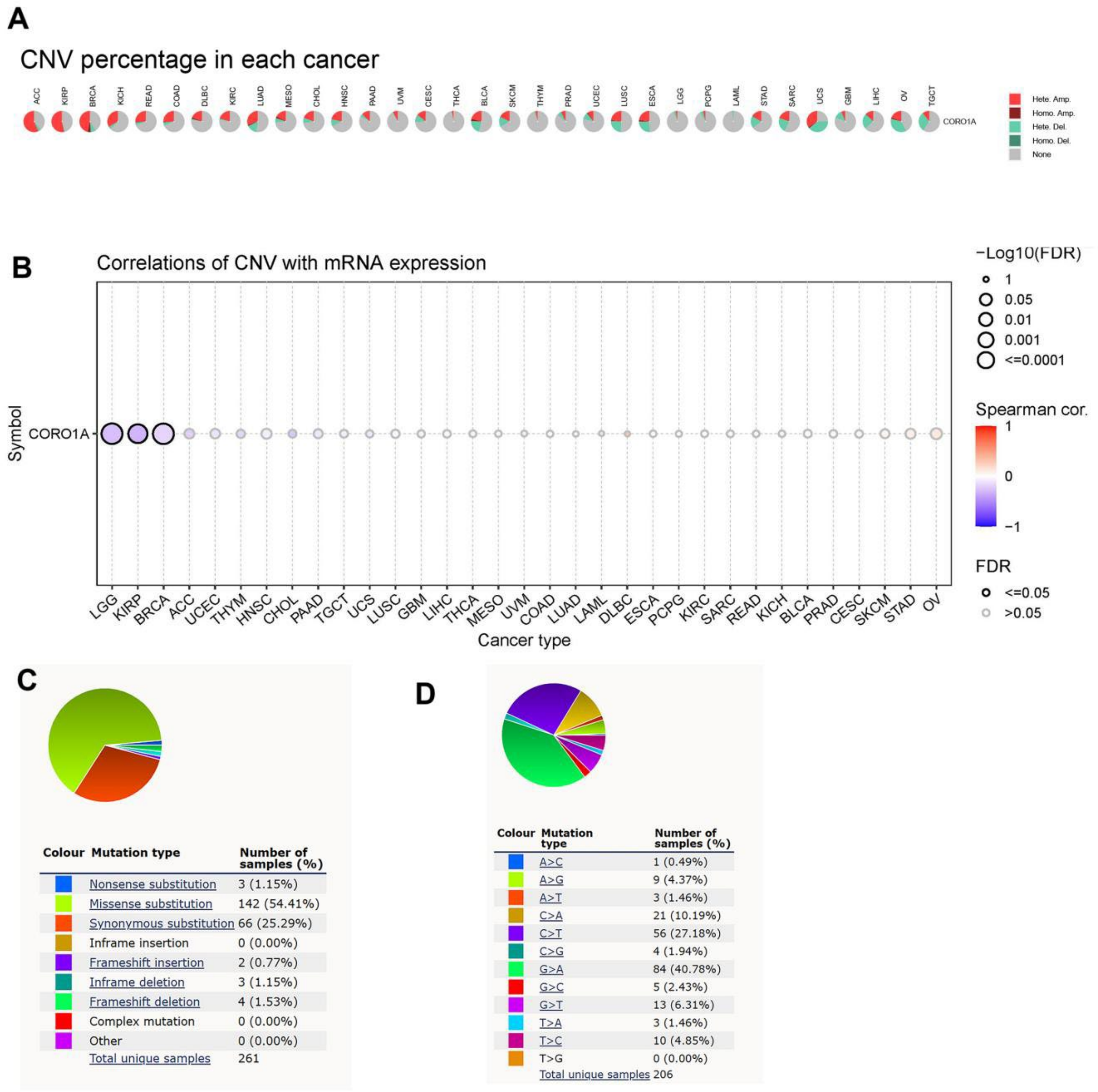

Fig.S3 Mutation analysis of CORO1A.**(A)** CORO1A copy number variant (CNV) percentage in each cancer. **(B)** The main mutation type of CORO1A. **(C)** The main type of single nucleotide variant (SNV) of CORO1A. **(D)** Correlation between CORO1A expression and CNV.

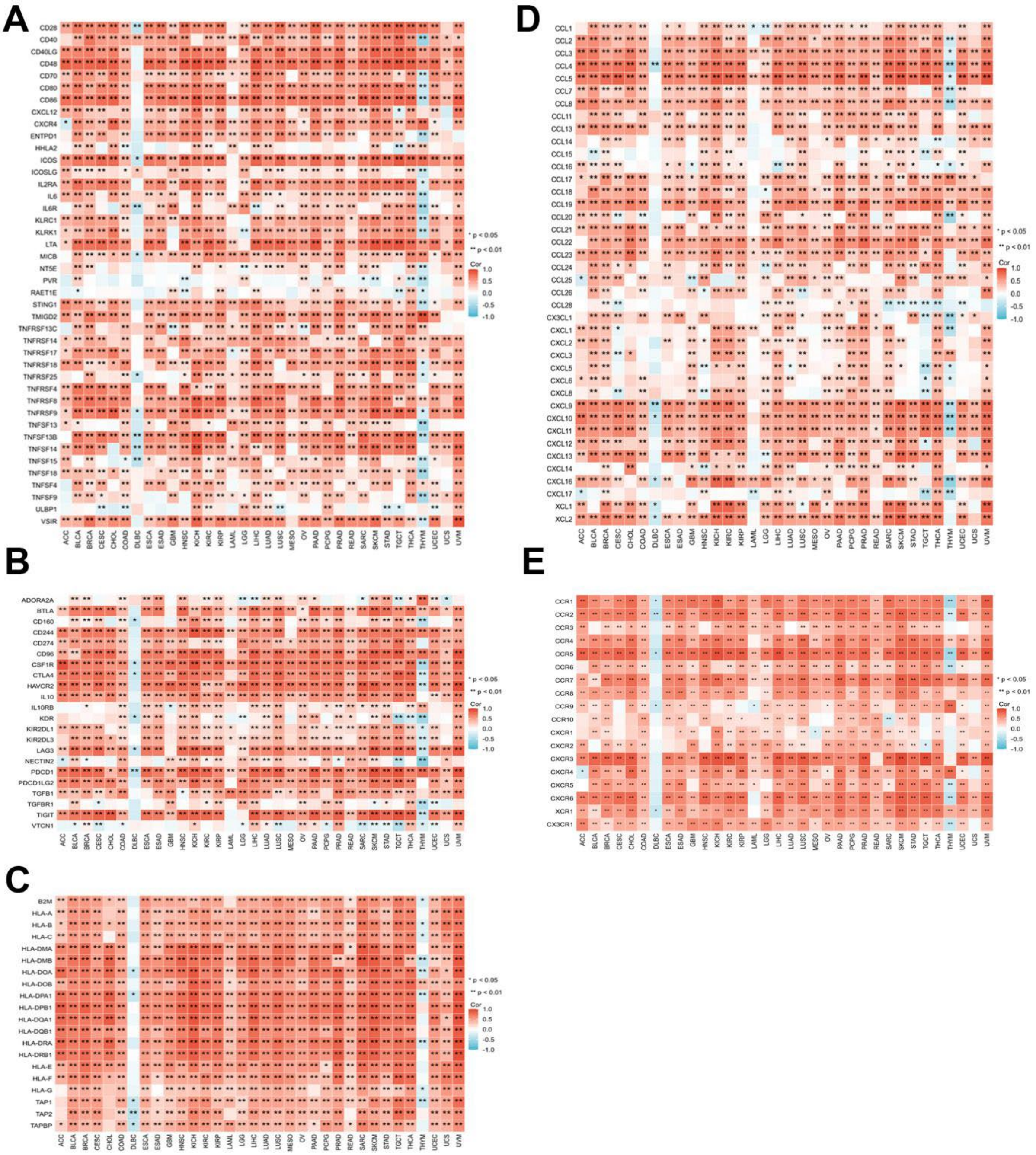

Fig.S4 CORO1A expression is associated with immune-associated genes in various cancers, including immunostimulatory genes **(A)**; immunoinhibitory genes **(B)**; chemokines **(C)**; chemokine receptors **(D)**; MHC genes **(E)**.

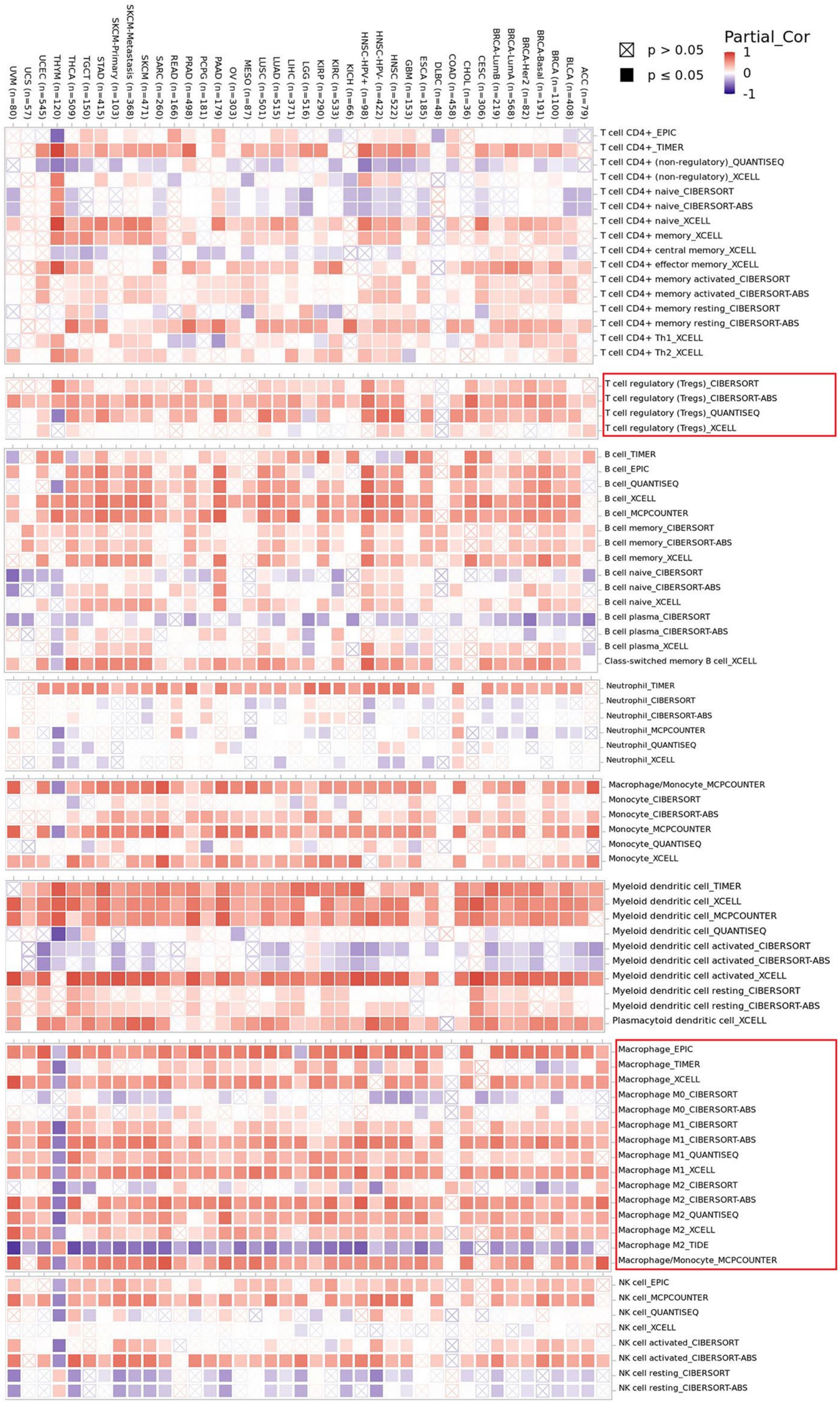

Fig.S5 Correlations between immune cell infiltration levels and CORO1A expression pan-cancer.

Supplementary Table.S1 Univariate and multivariate Cox analysis of clinical parameters in BRCA (A), CESC (B), HNSC (C), LGG (D), SKCM (E), UCEC (F).

A. BRCA

Characteristics	Total(N)	Univariate analysis		Multivariate analysis	
		Hazard ratio (95% CI)	P value	Hazard ratio (95% CI)	P value
Patholigic T stage	1065				
T1&T2	892	Reference		Reference	
T3&T4	173	2.091(1.447-3.020)	**<0.001**	1.079 (0.638-1.833)	**0.778**
Patholigic N stage	1049				
N0&N1	858	Reference		Reference	
N2&N3	191	2.385(1.628-3.495)	**<0.001**	1.244 (0.653-0.372)	**0.507**
Patholigic M stage	912				
M0	892	Reference			
M1	20	8.319(4.830-14.328)	**<0.001**	3.777（1.864-7.654）	**<0.001**
Patholigic stage	1044				
Stage I&Stage II	786	Reference			
Stage III&Stage IV	258	2.933(2.082-14.328)	**<0.001**	1.863（0.926-3.749）	**0.081**
Age	1068				
<=60	590	Reference			
>60	478	1.289 (0.810-4.132)	**<0.001**	1.041（0.709-1.527）	**0.838**
CORO1A	1068				
Low	534	Reference			
High	534	0.730 (0.344-0.885)	**0.014**	0.794（0.546-1.156）	**0.227**

***All P values presented in the table are in bold for legibility**

B. CESC

Characteristics	Total(N)	Univariate analysis		Multivariate analysis	
		Hazard ratio (95% CI)	P value	Hazard ratio (95% CI)	P value
Patholigic T stage	243				
T1&T2	212	Reference		Reference	
T3&T4	31	3.134(1.733-5.668)	**<0.001**	2.290 (0.689-7.609)	**0.176**
Patholigic N stage	195				
N0	134	Reference		Reference	
N1	61	2.108(1.062-4.187)	**0.033**	1.913(0.842-4.347)	**0.122**
Primary therapy outcome	219				
PD&SD	29	Reference			
PR&CR	190	0.110(0.064-0.190)	**<0.001**	0.226（0.084-0.605）	**0.003**
Clinical stage	299				
Stage I&Stage II	231				
Stage III&Stage IV	68	1.847(1.117-3.054)	**0.015**	0.186（0.036-0.973）	**0.046**
Age	306				
<=50	188	Reference			
>50	118	1.577 (0.993-2.504)	**0.053**	0.745（0.310-1.787）	**0.509**
CORO1A	306				
Low	153	Reference			
High	153	0.532 (0.331-0.855)	**0.009**	0.617（0.281-1.532）	**0.227**

C. HNSC

Characteristics	Total(N)	Univariate analysis		Multivariate analysis	
		Hazard ratio (95% CI)	P value	Hazard ratio (95% CI)	P value
Patholigic T stage	447				
T1&T2	179	Reference		Reference	
T3&T4	268	1.944(1.397-2.704)	**<0.001**	1.734 (0.942-3.189)	**0.077**
Patholigic N stage	410				
N0&N1	236	Reference		Reference	
N2&N3	174	2.109(1.533-2.902)	**<0.001**	1.719 (1.140-2.592)	**0.010**
Patholigic stage	435				
Stage I&Stage II	94	Reference			
Stage III&Stage IV	341	1.758(1.163-2.657)	**0.007**	1.080（0.469-2.485）	**0.856**
Clinical stage	489				
Stage I&Stage II	114				
Stage III&Stage IV	375	1.202(0.846-1.707)	**0.306**	0.822（0.432-1.564）	**0.550**
Primary therapy outcome	418				
PD&SD	47				
PR&CR	371	0.111(0.077-0.160)	**<0.001**	0.151（0.095-0.239）	**<0.001**
Gender	503				
Female	134				
Male	369	1.052(0.760-1.455)	**0.762**	0.927（0.619-1.387）	**0.712**
Age	503				
<=60	247	Reference			
>60	256	1.088 (0.820-1.444)	**0.559**	1.357（0.933-1.974）	**0.110**
Radiation therapy	441				
No	154				
Yes	287	0.843（0.610-1.166）	**0.303**	0.811（0.516-1.275）	**0.364**
CORO1A	503				

Characteristics	Total(N)	Univariate analysis		Multivariate analysis	
		Hazard ratio (95% CI)	P value	Hazard ratio (95% CI)	P value
Low	252	Reference			
High	251	0.705 (0.531-0.937)	**0.016**	0.855（0.588-1.245）	**0.414**

D. LGG

Characteristics	Total(N)	Univariate analysis		Multivariate analysis	
		Hazard ratio (95% CI)	P value	Hazard ratio (95% CI)	P value
WHO grade	468				
G2	223	Reference		Reference	
G3	245	1.609(1.192-2.172)	**<0.001**	1.221 (0.872-1.708)	**0.245**
IDH status	527				
WT	97	Reference		Reference	
Mut	430	0.192(0.141-0.261)	**<0.001**	0.249 (0.170-0.363)	**<0.001**
Primary therapy outcome	460				
PD&SD	257	Reference			
PR&CR	203	0.339(0.241-0.476)	**<0.001**	0.370（0.252-0.542）	**<0.001**
Gender	530				
Female	238				
Male	292	0.875(0.666-1.148)	**0.334**	0.905（0.665-1.230）	**0.522**
Age	530				
<=40	265	Reference			
>40	265	1.891 (1.432-2.498)	**<0.001**	1.876（1.355-2.597）	**<0.001**
CORO1A	530				
Low	264	Reference			
High	266	1.410 (1.070-1.858)	**0.015**	1.071（0.769-1.492）	**0.685**

E. SKCM

Characteristics	Total(N)	Univariate analysis		Multivariate analysis	
		Hazard ratio (95% CI)	P value	Hazard ratio (95% CI)	P value
Patholigic T stage	363				
T1&T2	119	Reference		Reference	
T3&T4	244	1.681(1.281-2.205)	**<0.001**	1.487 (1.101-2.009)	**0.010**
Patholigic N stage	404				
N0&N1	299	Reference		Reference	
N2&N3	105	1.874(1.434-2.451)	**<0.001**	1.584(1.050-2.390)	**0.029**
Patholigic M stage	432				
M0	408	Reference			
M1	24	2.033(1.260-3.282)	**0.004**	1.521（0.756-3.058）	**0.240**
Patholigic stage	412				
Stage I&Stage II	218	Reference			
Stage III&Stage IV	194	1.744(1.372-2.218)	**<0.001**	1.459（1.026-2.073）	**0.035**
Gender	458				
Female	173				
Male	285	1.046(0.828-1.321)	**0.705**	1.046（0.792-1.382）	**0.750**
Age	458				
<=65	248	Reference			
>65	210	1.585 (1.247-2.013)	**<0.001**	1.267（0.951-1.688)	**0.105**
CORO1A	458				
Low	228	Reference			
High	230	0.771 (0.616-0.965)	**0.023**	0.739（0.564-0.969）	**0.029**

F. UCEC

Characteristics	Total(N)	Univariate analysis		Multivariate analysis	
		Hazard ratio (95% CI)	P value	Hazard ratio (95% CI)	P value
Clinical stage	553				
Stage I&Stage II	394	Reference			
Stage III&Stage IV	159	3.183(2.251-4.500)	**<0.001**	3.341（2.262-4.935）	**<0.001**
Primary therapy outcome	482				
PD&SD	26				
PR&SR	456	0.100(0.063-0.160)	**<0.001**	0.134（0.082-0.218）	**<0.001**
Age	551				
<=60	207	Reference			
>60	344	1.356 (0.936-1.966)	**0.107**	1.147（0.758-1.735）	**0.517**
Radiation therapy	529				
No	281				
Yes	248	1.102 (0.776-1.566)	**0.588**	1.013（0.689-1.489）	**0.949**
CORO1A	553				
Low	277	Reference			
High	276	0.545 (0.380-0.782)	**0.001**	0.602（0.403-0.898）	**0.013**

Table.S2 The top 100 CORO1A-associated genes.

Table.S3 GO terms and KEGG pathways enriched.
