# Supplementary material for: CORO1A: a pan-cancer prognosis, diagnostic and immune biomarker based on breast cancer validation
Source: Front Oncol. 2025 Oct 6;15:1670526. doi: 10.3389/fonc.2025.1670526 (PMC12535887; doi:10.3389/fonc.2025.1670526)
Supplement: Supplementary file 2 [file DataSheet2.pdf]

# Supplementary materials for

# A Comprehensive Assessment of CORO1A's Role as a Prognostic, Diagnostic, and Immune Biomarker in Multiple Cancer Types

**Dilraba Elihamu[1†], Yongxiang Li[1†], Yiyang Wang[1†], Haiyan Cui[2], Yiting Xing[1], Haohao Peng[1], Dilimulati Ismtula[1], Chenming Guo[1*]**

1 Department of Breast Surgery, Center of Digestive and Vascular Surgery, The First Affiliated Hospital of Xinjiang Medical University, Urumqi, China

2 Bayingolin Mongolian Autonomous Prefecture People's Hospital, Korla City, Xinjiang Uygur Autonomous Region, 841000, China

*Corresponding author.

† These authors contributed equally to this work and shared the first authorship.

*Correspondence: Chenming Guo, gcm_xjmu@yeah.net.

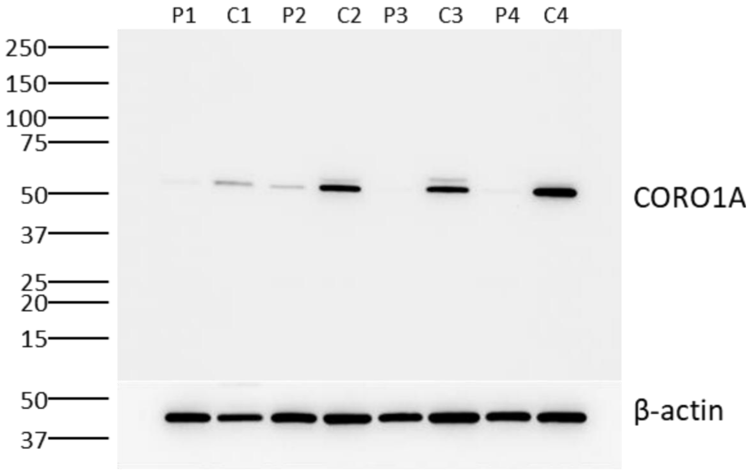
P1 C1 P2 C2 P3 C3 P4 C4
250
150
100
75
50
37
25
20
15
CORO1A
50
37
β-actin

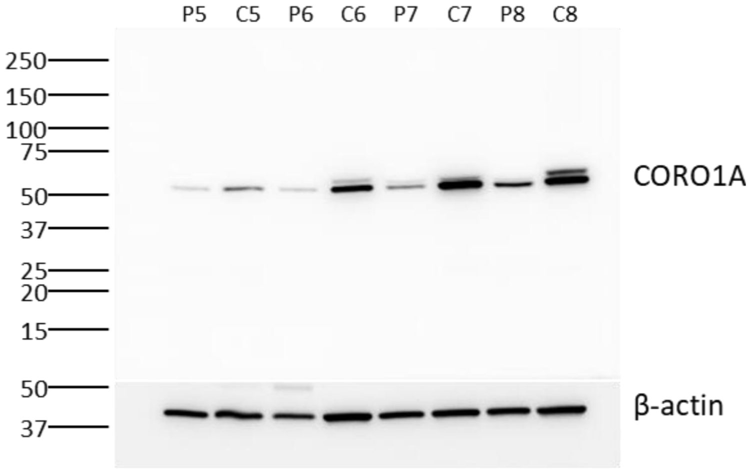
P5 C5 P6 C6 P7 C7 P8 C8
250
150
100
75
50
37
25
20
15
CORO1A
50
37
β-actin

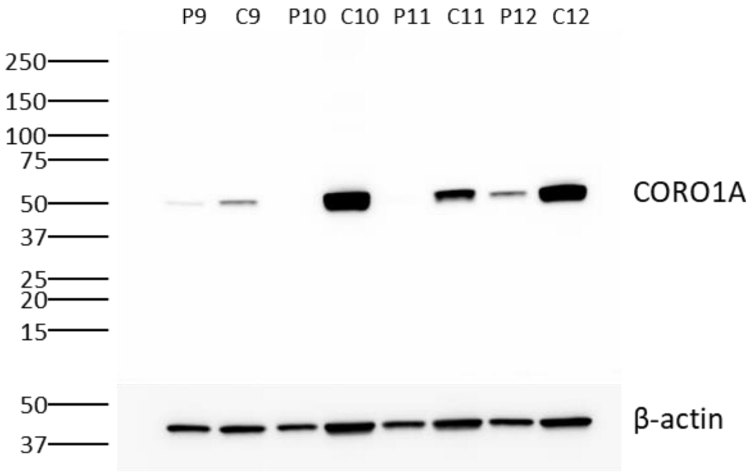
P9 C9 P10 C10 P11 C11 P12 C12
250
150
100
75
50
37
25
20
15
CORO1A
50
37
β-actin

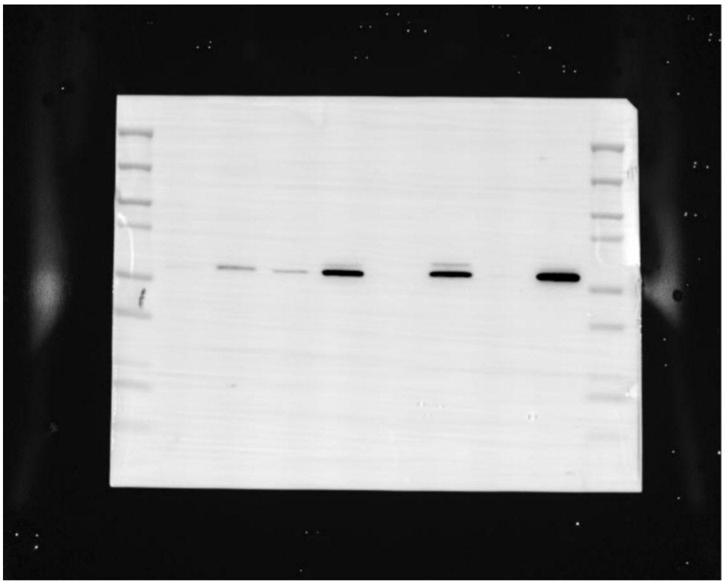

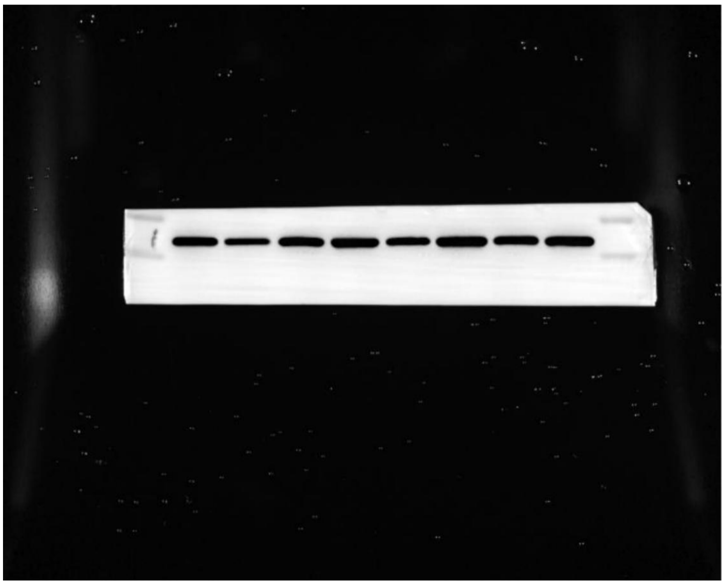

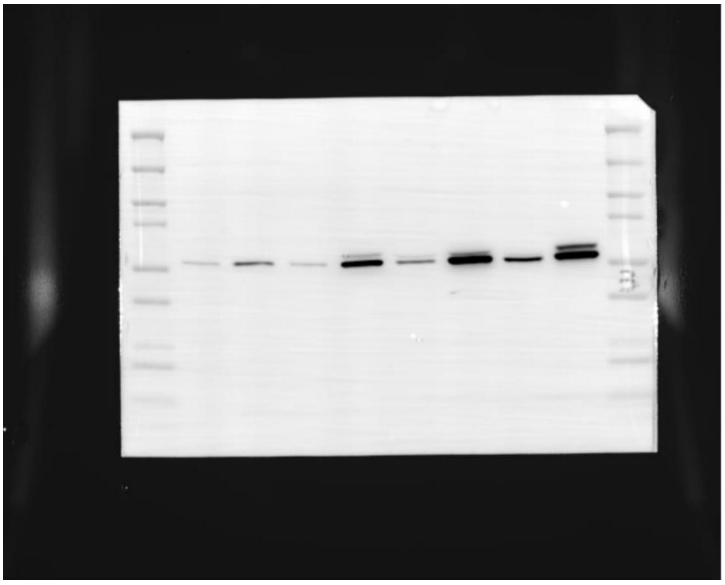

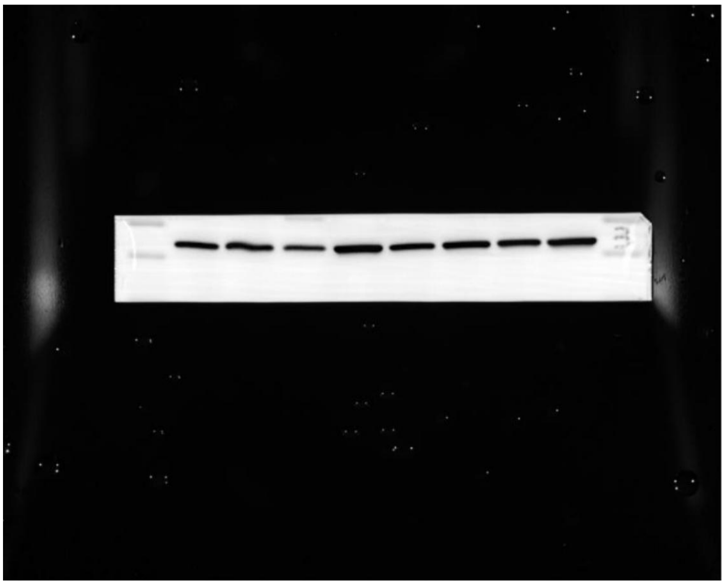

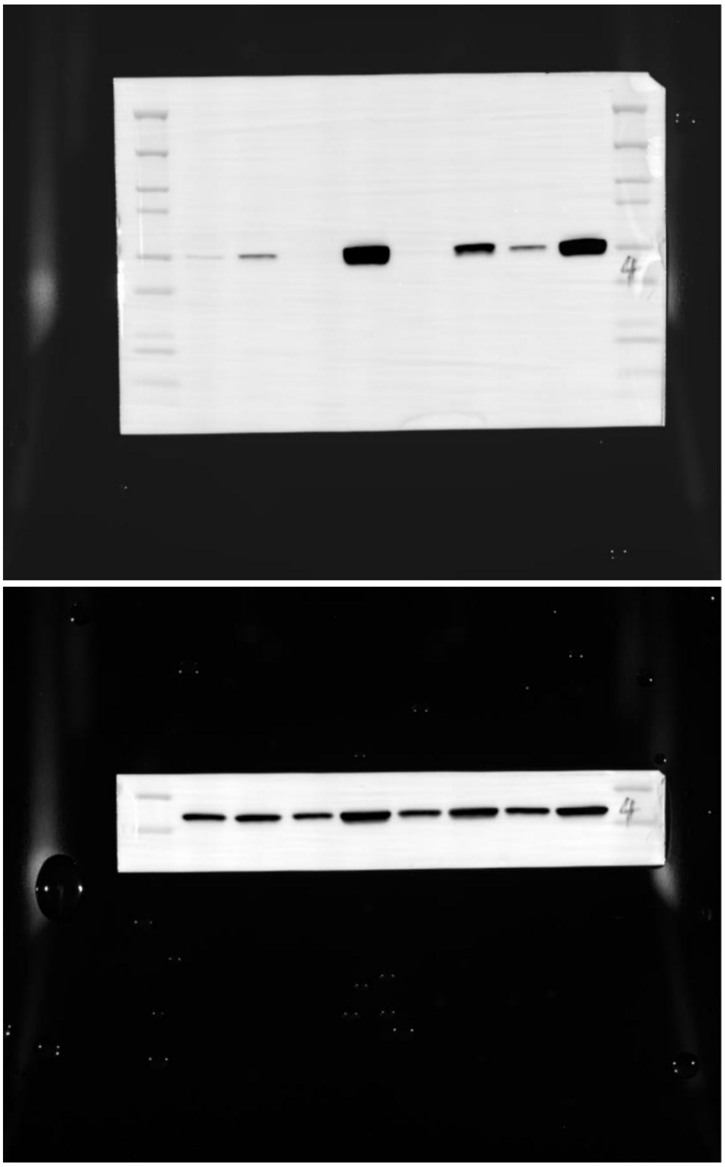

**Fig.S6** Original Western blot whole membranes of 12 breast cancer patients and adjacent tissues.

## MCF-7

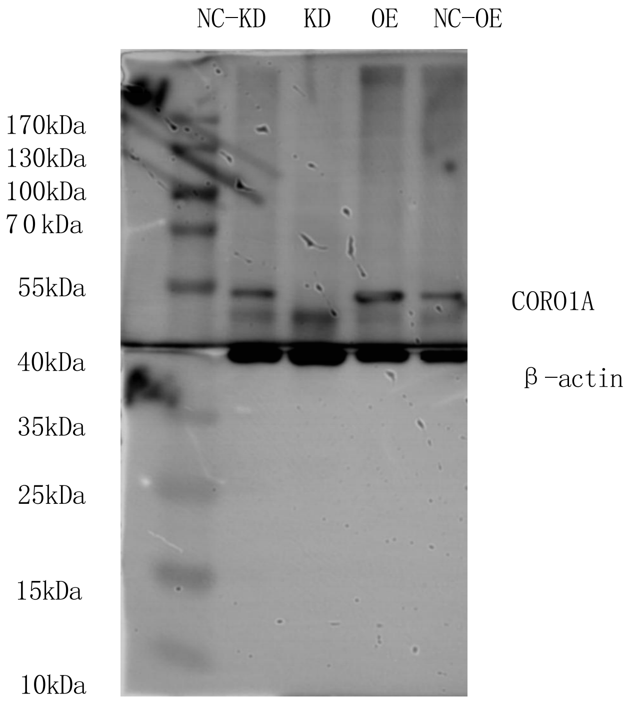


## MDA-MB-231

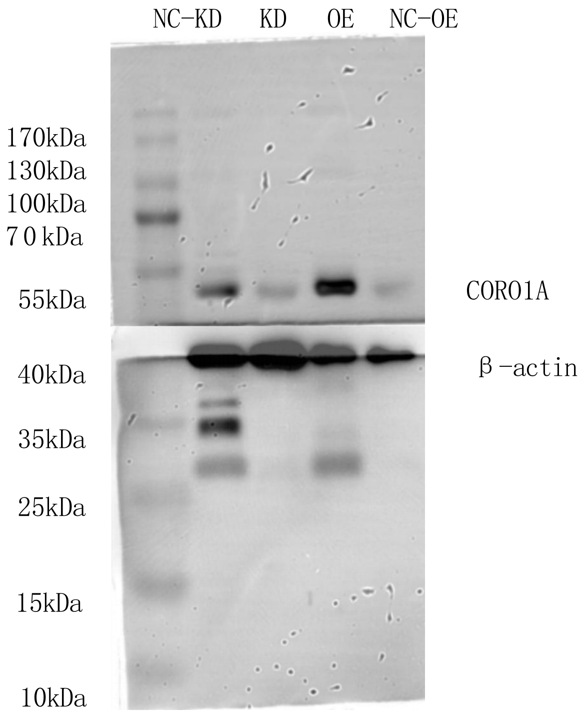


**Fig.S7** Western blot original images of CORO1A knockdown or overexpression in breast cancer cell lines.
